# Pancreatic α cells are required for nutrient homeostasis by regulating dynamic β cell networks in islets

**DOI:** 10.1126/sciadv.aea9045

**Published:** 2026-07-03

**Authors:** Marie Lallouet, Manon Jaffredo, Antoine Pirog, Karen Leal-Fischer, Julien Gaitan, Daniel T. Meier, Sylvie Renaud, Matthieu Raoux, Jochen Lang

**Affiliations:** ^1^ Univ. Bordeaux, CNRS, Bordeaux INP, CBMN, UMR 5248, F-33600 Pessac, France.; ^2^ Electronics-Physics-Acoustics Department, Junia, F-59800 Lille, France.; ^3^ Department of Biomedicine, University of Basel, F-33400 Basel, Switzerland.; ^4^ Univ. Bordeaux, CNRS, Bordeaux INP, IMS, UMR 5218, F-33400 Talence, France.

## Abstract

Pancreatic islets contain α, β, γ, and δ cells as sensors and actuators regulating glucose homeostasis. Despite the known importance of α cells, they are seemingly required for glucose tolerance only under metabolic stress. In an inducible model of α cell ablation in mice (GluDTR), glucose tolerance was considerably decreased by the addition of amino acids mimicking meals. Analysis of islet β cell secretion and electrical activities using microelectrode arrays (MEAs) detected only minor differences in GluDTR mice for glucose but revealed a major reduction upon addition of amino acids. Analysis of functional islet β cell networks by high-density MEA revealed leader regions in different locations, a high degree of synchrony, and the activation of large cell clusters. The characteristics of leading regions were preserved in GluDTR islets, but synchrony, cluster size, and signal propagation speed were largely reduced. Thus, even without metabolic stress, α cells are required for nutrient homeostasis by regulating the dynamics of β cell networks.

## Teaser

Islet α cells are required for meal tolerance by tuning synchrony, cluster size, and signal propagation of β cell networks.

## INTRODUCTION

Pancreatic islets play a major role in nutrient homeostasis, and their dysfunction leads to the different forms of diabetes (*1*). These micro-organs consist of four major cell types: The insulin-secreting β cells and proglucagon-derived peptides [glucagon and glucagon-like peptide–1 (GLP-1)] secreting α cells release hormones into the systemic circulation, whereas somatostatin secreted from δ cells and the less explored γ cells are thought to have a role mainly within the islets (*2*). Islet cells have the simultaneous role of actuators and sensors: Whereas β cells mainly sense glucose, α cells are important for glucose and amino acid detection (*3*). The adaptive function of islets requires cross-talk between the different cell types (*4*), and secretory activities of α and β cells are known to oscillate in a phase-locked fashion in vivo and in vitro upon glucose or nutrient stimulation (*5*–*7*). Thus, although, during stimulation, the overall secretion of α cell–derived peptides is less than at low glucose, islet β cells are exposed to repetitive pulses of these peptides, and they may influence β cell activity under stimulatory conditions. Islet α cells release glucagon and a minor amount of GLP-1 (*8*–*11*). Whereas glucagon can activate both glucagon and GLP-1 receptors, GLP-1 is specific and far more potent for its cognate receptor (*10*). Both receptors activate downstream adenylyl cyclases as well as phospholipases (*12*–*14*), and the effects of glucagon as well as GLP-1 on insulin secretion from β cells are mediated by both the adenosine 3′,5′-monophosphate (cAMP)–dependent and cAMP-independent pathways (*15*, *16*).

Simulations of α-β cross-talk predict that a specific model, where α cells activate β cells and β cells inhibit α cells, results in the most harmonic response to changes in glucose concentrations with improved glucose tolerance and amino acid handling (*17*, *18*). Nevertheless, the precise interaction between islet cell types under various nutrient stimulations is still not fully elucidated. Mouse models with ablation of a given cell type via specific expression of the diphtheria toxin receptor (DTR) and treatment with diphtheria toxin (DT) have provided information on cell type–specific in vivo interaction. The deletion of α cells (GluDTR) did not significantly alter glucose tolerance in young mice and led to an impairment only upon metabolic stress or aging (*19*–*21*), similar to the observations in mice lacking the β cell glucagon and GLP-1 receptors (*8*, *22*). Moreover, deleting all cell types except for β cells improved glucose tolerance in vivo with normal insulin secretion in vitro (*23*). In vitro analysis of GluDTR mice showed reduced in vitro insulin secretion in response to glucose that could be restored by addition of exogeneous glucagon, GLP-1, or the adenylyl cyclase activator forskolin (*10*, *22*). Similarly, the effect of amino acids on insulin secretion was diminished in vitro in a mouse model where the α cell activity was either blunted by activation of an inhibitory designer heterotrimeric guanine nucleotide–binding protein–coupled receptor (*24*) or when the glucagon receptor was absent on β cells (*8*).

Amino acids account for one-third of the calories in a standard meal, and they increase insulin secretion in humans and lower blood glucose (*25*). In recent years, the awareness has increased about the role of α cells as sensors for amino acids (*26*), and the influence of amino acids on the secretory activity of β cells via α cells has been established in vitro (*24*). However, their effect on cellular or network properties have not been addressed. β cells are electrogenic, and their increase in metabolism leads to changes in plasma membrane ion fluxes, resulting in insulin exocytosis via Ca^2+^-dependent processes (*27*). Activation of islet β cells implies dynamic networks and functional coupling among them for an optimal physiological response (*28*). It remains largely unknown how these membrane ion fluxes and electrical networks are modulated by α cells during the long postprandial period. Extracellular electrophysiology offers a convenient means for online, noninvasive, and long-term monitoring and does not require biasing methods such as loading of fluorescent agents or manipulations of the sample for genetically encoded sensors. Moreover, electrophysiology offers a temporal resolution unmatched by other approaches. Microelectrode arrays (MEAs) measure changes in field potentials and intercellular coupling. Coordination between islet β cells, a hallmark of islet activation, can be reliably detected and analyzed by the monitoring of so-called slow potentials (SPs) without bias (*29*–*36*).

We have now used this approach to characterize the roles of glucose and amino acids in β cell activation, their network dynamics, and the role of α cells herein using acute α cell–specific deletion by DTR expression and toxin treatment (*19*, *21*, *22*). Moreover, we developed the use of high-density MEA (HD-MEA) for the fine analysis of β cell activation in islet subregions and to establish the characteristics of physiological β cell networks in the presence and absence of α cells. Our in vivo characterization and islet analysis demonstrate a major role for α cells in the β cell response to amino acids in terms of glucose tolerance, islet activity, and β cell network properties.

## RESULTS

### α cells mediate the effect of amino acids on glycemia and insulinemia

**Fig. 1.**
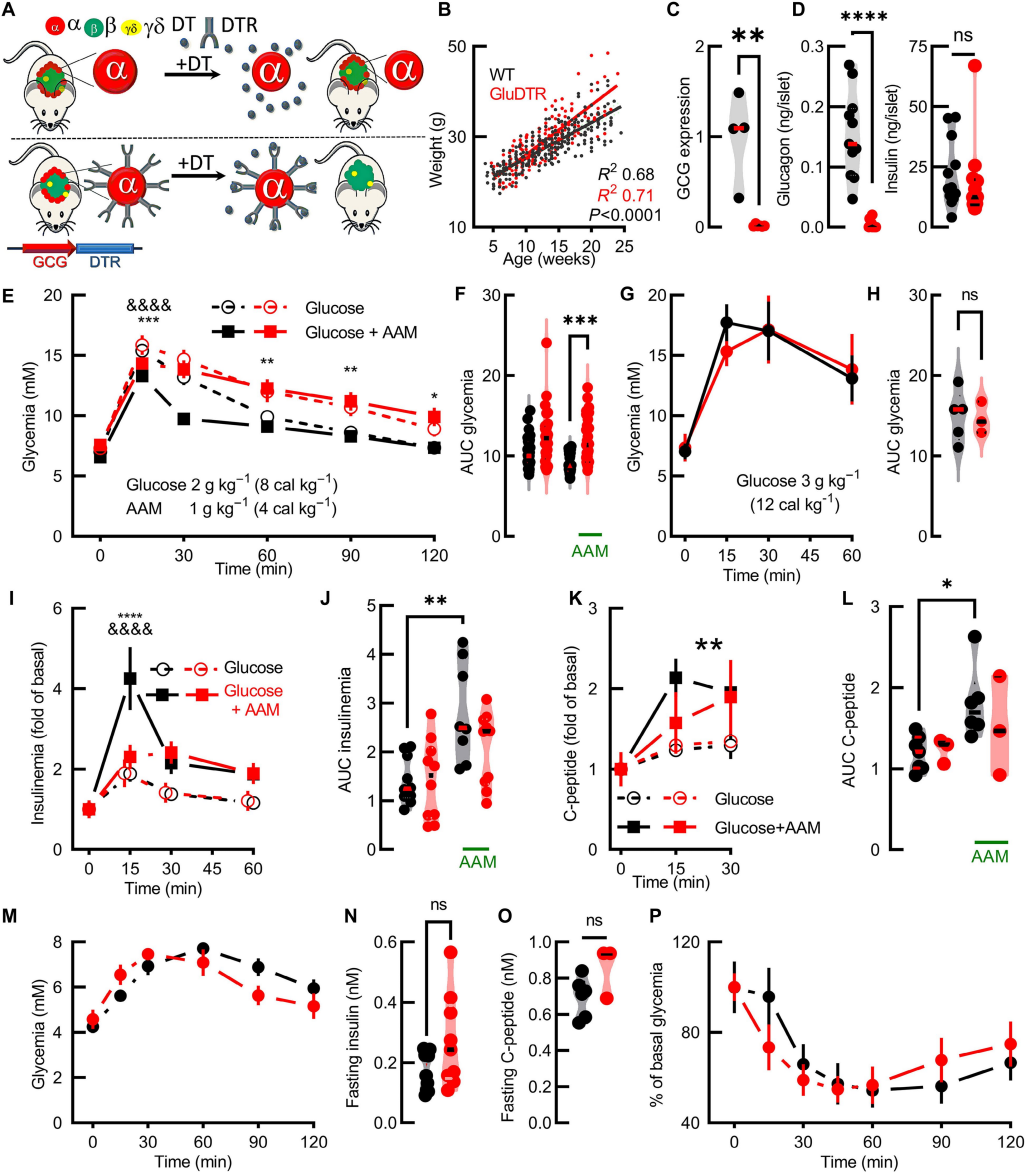
Animal model and in vivo characterization of WT and GluDTR mice. WT data in black and GluDTR data in red. (**A**) GluDTR mice expressing the human DTR under the control of the rat glucagon promoter were fully backcrossed to a C57BL/6N genetic background. Injection of DT at 5 to 8 weeks of age leads to selective destruction of α cells. (**B**) Growth characteristics in terms of weight. *N* = 31 to 33. (**C**) Islet glucagon expression. *N* = 4. (**D**) Islet hormone contents. *N* = 10 to 11. (**E**) Intraperitoneal glucose and amino acid tolerance test in WT and GluDTR mice (2 g/kg glucose and 1 kg/g amino acids). *N* = 24 to 30. (**F**) Area under the curve (AUC) of (D). (**G**) Intraperitoneal glucose tolerance test in WT and GluDTR mice (2 g/kg glucose). *N* = 3 to 5. (**H**) AUC of (F). (**I** and **J**) Insulinemia and AUC during the intraperitoneal glucose and amino acid tolerance test [see (E) and (F)]. *N* = 8 to 10. (**K** and **L**) C-peptide levels and AUC during the intraperitoneal glucose and amino acid tolerance test [see (D) and (E)]. *N* = 3 to 7. (**M**) Pyruvate tolerance test (2 g/kg pyruvate intraperitoneally). *N* = 7 to 8. (**N** and **O**) Fasting insulin and C-peptide levels. *N* = 9 to 10. (**P**) Insulin tolerance test (0.5 U/kg). *N* = 9 to 10. *2*P* < 0.05, **2*P* < 0.01, ***2*P* < 0.001, and ****2*P* < 0.0001; ns, not significant; in (D) and (H): **** or ***, WT glucose and AAM as compared to WT glucose alone; ^&&&&^2*P* < 0.0001, WT as compared to glucose and AAM in GluDTR; (C) and (D), Mann-Whitney; (E) to (L), one- or two-way analysis of variance (ANOVA) or Tukey or Holm-Sidak post hoc test.

To obtain mice with islets lacking α cells, we used a genetic mouse model of inducible α cell ablation subsequent to cell-specific expression of the human DTR under the control of a glucagon promoter (*19*, *21*, *22*), termed GluDTR. Injection of DT leads to progressive loss of α cells (Fig. 1A). Induced GluDTR mice gained some more weight as compared to the wild type (WT) (Fig. 1B). Islet hormone analysis confirmed a large reduction in glucagon gene expression and content in GluDTR mice, whereas insulin contents did not differ (Fig 1, C and D). Intraperitoneal glucose tolerance tests demonstrated no difference between WT and GluDTR mice when injecting glucose only (Fig. 1, E and F). Adding a mix of 19 amino acids to glucose led to a significant reduction of glycemia in WT mice, and the areas under the curve during the first 30 min were significantly different (28.6 ± 1.2 versus 23.0 ± 0.7; Tukey 2*P* < 0.01). This glycemia-lowering effect of amino acid mix (AAM) was largely but not completely absent in GluDTR mice as meal-mimicking mixtures also contain amino acids stimulating mainly β cells (Fig. 1E) (*37*). To rule out the notion that this was due to an increase in calories injected, we also tested intraperitoneal glucose tolerance at the same number of calories as glucose and AAM combined, i.e., 3 g/kg glucose (Fig. 1, G and H). Under this condition, glucose tolerance in WT mice again resembled that of GluDTR mice, ruling out calories as the responsible factor. As the presence of AAM considerably improved glucose tolerance in WT mice during the first 30 min, we also measured blood insulin and C-peptide levels (Fig. 1, I to L). After intraperitoneal injection of glucose alone, there was no difference in insulin levels between WT and GluDTR mice. The presence of AAM considerably increased hormone levels in WT animals but had no effect on GluDTR mice. Pyruvate tolerance tests provide some crude means to assess the role of liver in glucose homeostasis (*38*), and glucagon regulates gluconeogenesis (*9*). This test did not show any difference between WT and GluDTR mice (Fig. 1M). Moreover, fasting insulin or C-peptide levels or insulin tolerance test did not show any difference (Fig. 1, N and O). Thus, the main difference in vivo between WT and GluDTR animals resides in the glycemic response to amino acids in the presence of glucose. Last, there was no significant difference between WT and GluDTR mice during the insulin-tolerance test (Fig. 1P).

### α cells enhance β cell electrical activity mainly during the second phase in the presence of AAM

**Fig. 2.**
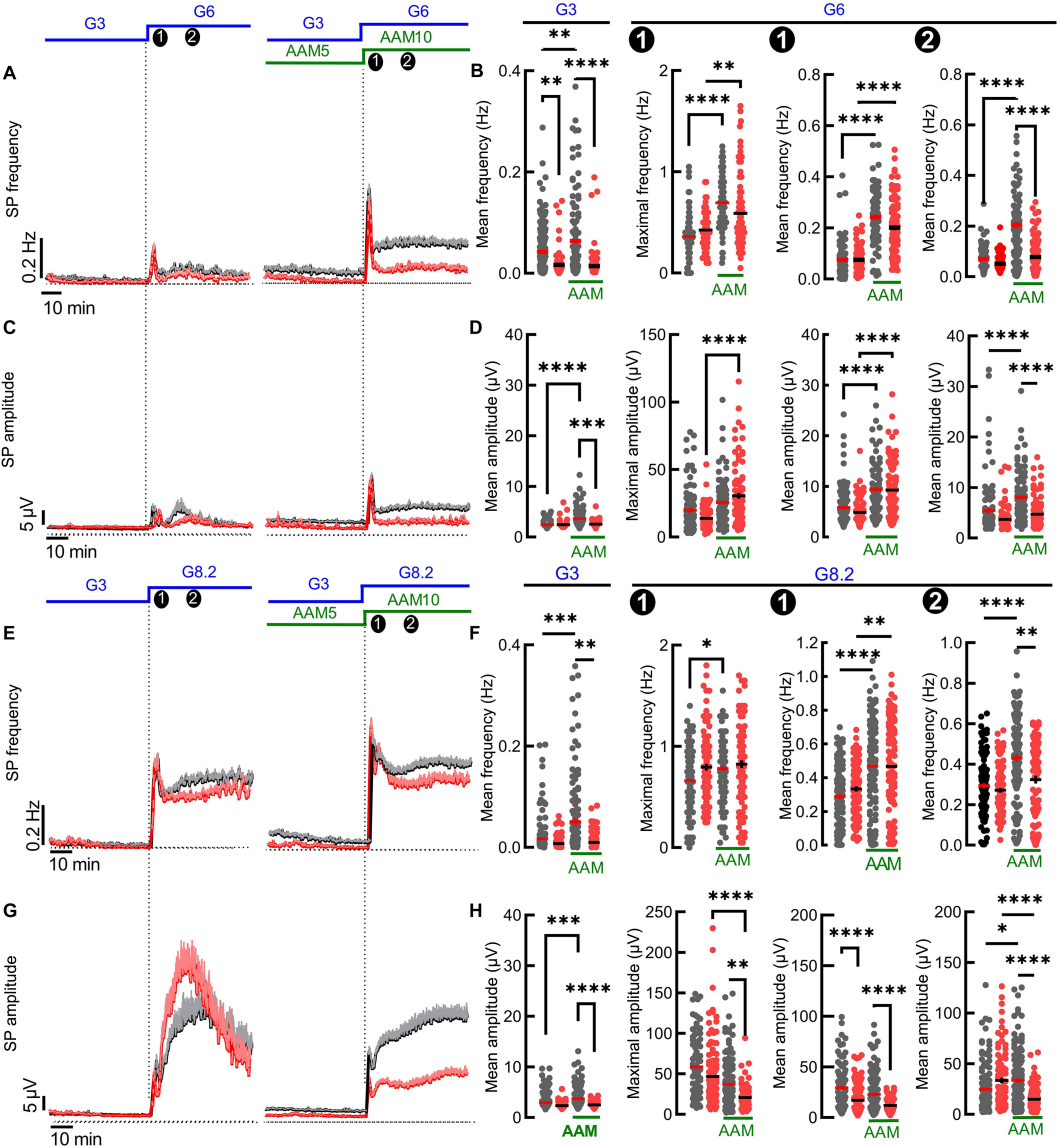
Effects of amino acids on glucose-evoked electrical activity in islets recorded by MEAs. WT data in black and GluDTR data in red. (**A**) SP mean frequencies (±SEM) evoked by G3 followed by G6 in the absence or presence of 5 and 10 mM AAM (AAM5 and AAM10, respectively), respectively. First and second phases are indicated (① and ②, respectively). *N* = 3 to 4; *n* = 98 to 110. (**B**) Statistics of (A) (G3; ①, 0 to 300 s; ②, 700 to 2700 s after the increase of glucose) and maximal frequencies of the first phase. (**C** and **D**) SP amplitudes and statistics as in (A) and (B). *N* and *n* as in (A) and (B). (**E** and **F**) Same as (A) and (B), with mean SP frequencies but using G8.2 as the stimulatory glucose concentration. *N* = 7; *n* = 99 to 134. (**G** and **H**) Same as (C) and (D), with mean SP amplitudes but using G8.2 as the stimulatory glucose concentration. *N* = 7; *n* = 99 to 134. ANOVA and Kruskal-Wallis; *2*P* < 0.05, **2*P* < 0.01, ***2*P* < 0.001, and ****2*P* < 0.0001.

Extracellular electrophysiology, as done here using MEAs, provides direct information on the electrical activity of islets, and the amplitude of so-called SPs provides an excellent measure of islet β cell synchrony through their electrical coupling (*31*, *34*). The multicellular SPs represent summations of synchronized intracellular slow plateau depolarizations of β cells. An increase in their amplitude reflects an increased temporal overlap of bursts resulting from enhanced synchrony. We have used this approach here to evaluate the effect of different glucose concentrations, either alone or in combination with AAM, on islets (Fig. 2). We choose 3 mM glucose (G3) concentrations as basal, 6 mM glucose (G6) as intermediary where islets start to respond, and 8.2 mM glucose (G8.2), near the maximum effective concentration of 10 mM (*34*). At G3, GluDTR islets were slightly less active in terms of frequency, and this was further accentuated in the presence of glucose and AAM. G6 induced clear first and second phases in the frequency and amplitude of SPs (Fig. 2, A and D) without any significant differences between WT and GluDTR islets (Fig. 2, C and F).

In contrast, the addition of AAM to G6 revealed clear differences in WT versus GluDTR islets (Fig. 2, B and E). The presence of AAM increased, in both WT and GluDTR, the mean frequency and amplitude during glucose stimulation in the first and second phases. Notably, the increase in the second-phase mean frequency and amplitude was far more pronounced in the WT islets and significantly different from GluDTR islets (Fig. 2, B, C, E, and F), the latter arising to only 38% (frequency) and 59% (amplitude) of WT islet values. We next examined the effect of a slightly higher glucose concentration, 8.2 mM. Overall, frequencies and amplitudes were increased as compared to G6. Again, frequencies and amplitudes were comparable between WT and GluDTR islets (Fig. 2, G and J). In the presence of AAM and G8.2 (Fig. 2, H and K), differences were apparent between WT and GluDTR islets in the mean frequencies of the second phase and even more pronounced in mean amplitudes during the second phase (Fig. 2, I and L), which amounted in the GluDTR islets only to 45% of those in the WT. Thus, similar to in vivo data, the presence of amino acids induced a pronounced difference between the WT versus GluDTR and was present mainly during the second phase.

**Fig. 3.**
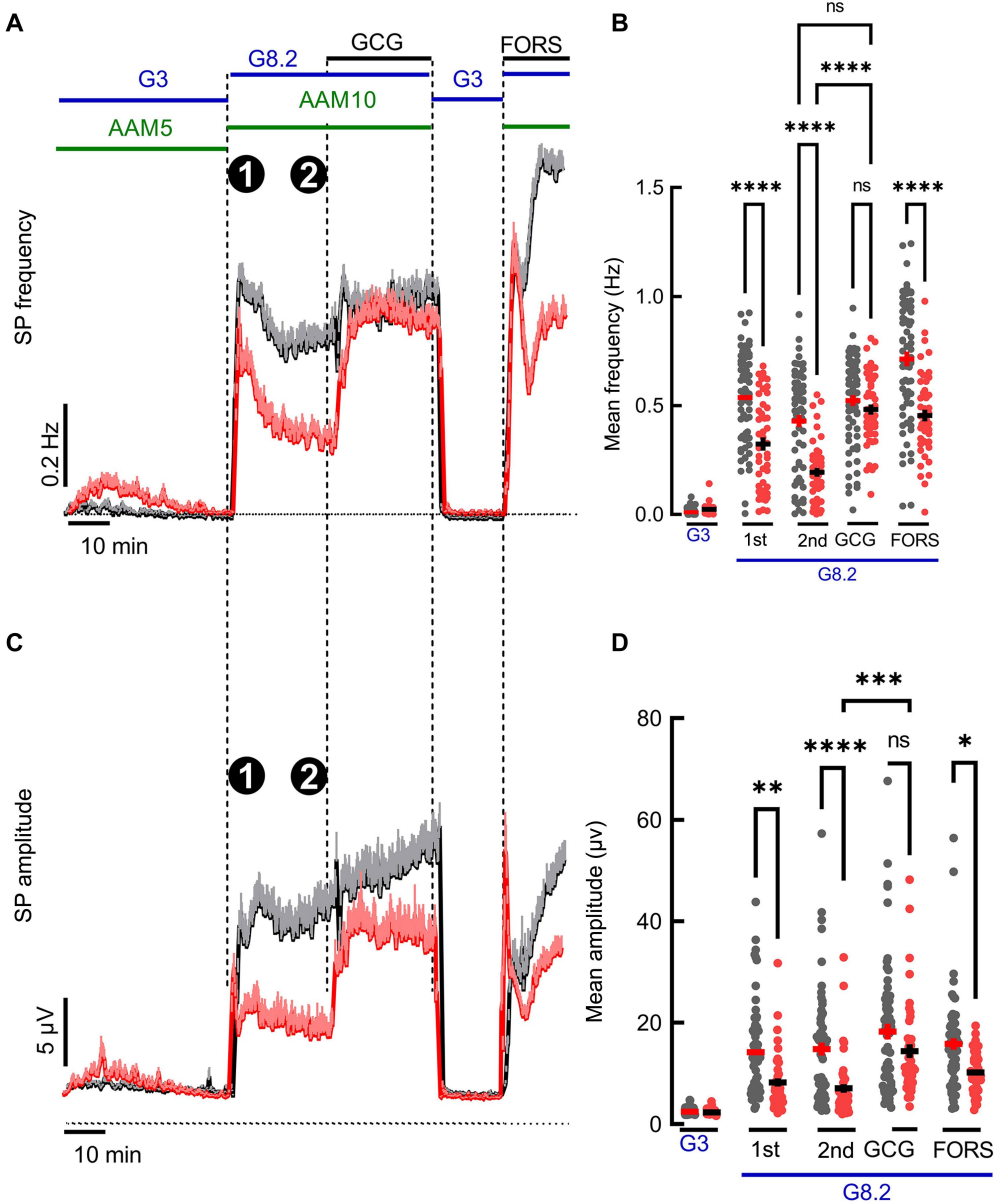
Effect of glucagon and forskolin on electrical activity stimulated by glucose and amino acids in WT and GluDTR islets. (**A**) Effect of glucose (G3 and G8.2), amino acids (AAM5 and AAM10), glucagon (GCG; 1 nM) or forskolin (FORS; 1 μM) on mean SP frequencies (±SEM). First and second phases are indicated (① and ②, respectively). (**B**) Statistics of mean frequencies. *N* = 4; *n* = 49 to 68. (**C**) Effect of glucose, amino acids, glucagon, or forskolin [concentrations as in (A)] on mean SP amplitudes (±SEM) (① and ②). (**D**) Statistics of mean amplitudes. Two-way ANOVA and Tukey; *2*P* < 0.05, **2*P* < 0.01, ***2*P* < 0.001, and ****2*P* < 0.0001.

As the lack of effects of AAM in the absence of α cells is most likely due to the absence of α cell hormones, we tested next whether exogenous glucagon can restore the activity of GluDTR islets. To this end, we added glucagon to the second phase during stimulation of islets by G8.2 and AAM (Fig. 3). We again observed a marked difference between WT and GluDTR islets upon exposure to glucose and amino acids, especially during the second phase (Fig. 3, A and C). The addition of exogeneous glucagon (Fig. 3, A to D) induced recovery by increasing the second-phase frequencies from 45 ± 5% of WT (both without glucagon) to 92 ± 4% of WT (both with glucagon). Amplitudes were recovered to a slightly lesser degree from 48 ± 6% of WT (without glucagon) to 80 ± 7% of WT (both with glucagon). In contrast, forskolin, an activator of adenylyl cyclases, increased both frequencies and amplitudes in GluDTR islets but only to 64 ± 4% of WT islets. We also tested for the contribution of GLP-1 receptors in WT islets using a relatively stable antagonist, exendin 9-39, to avoid tissue degradation of GLP-1 and the potential generation of antagonist intermediates (*39*). The presence of exendin 9-39 considerably reduced the amplitude and frequency mainly throughout the second phase (fig. S1), suggesting a role for GLP-1 receptors in the electrical activation of islets by glucose and amino acids.

**Fig. 4.**
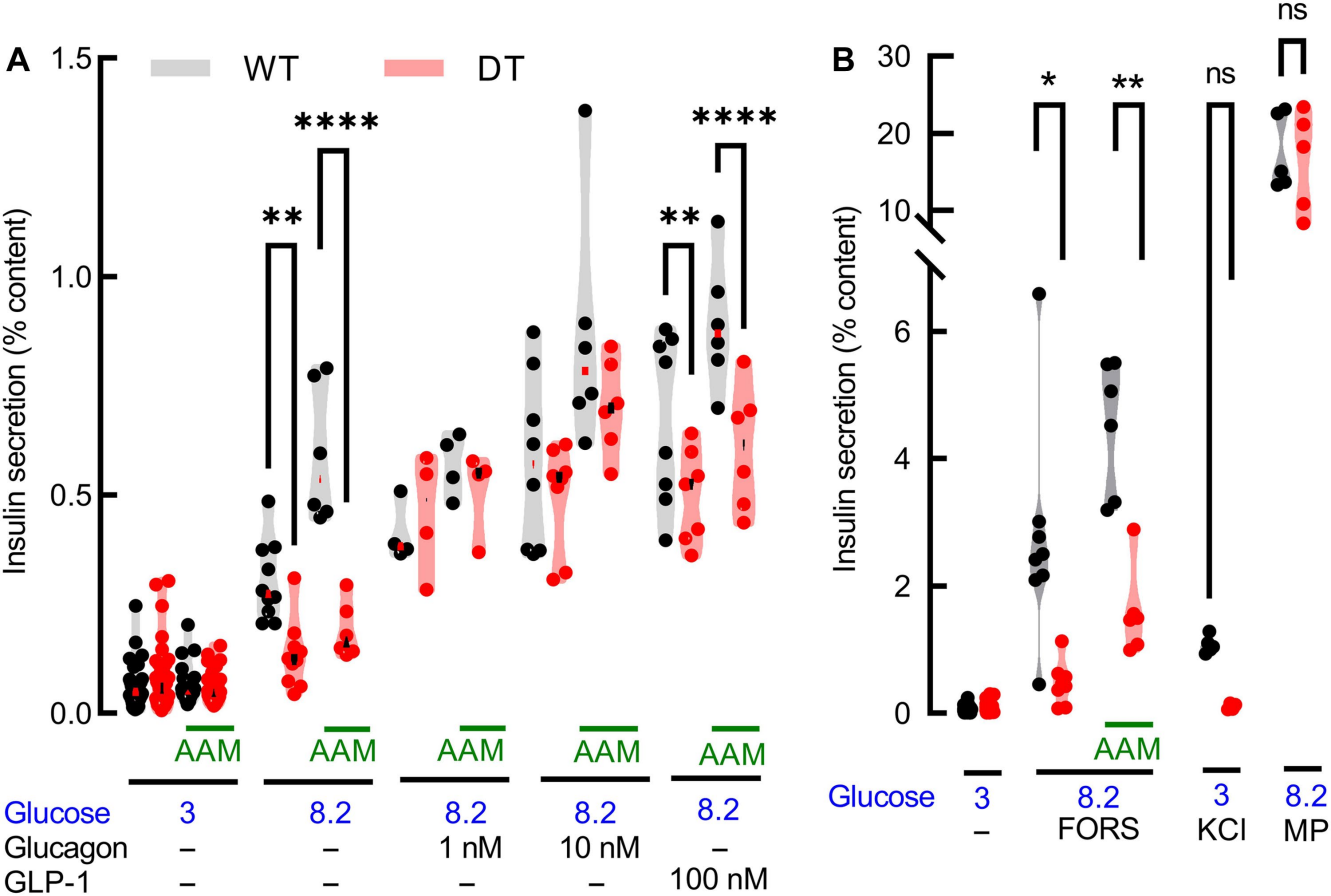
Insulin secretion from WT or GluDTR islets. (**A**) During secretion assays, islets were incubated with G3 or G8.2 in the absence or presence of amino acids (AAM; 5 mM at G3 and 10 mM at G8.2) and in the absence or presence of glucagon or GLP-1. *N* = 3 to 6; *n* = 12 to 77. (**B**) Islets were incubated in the absence or presence of amino acids (AA; 10 mM) with G3 or G8.2 and in the absence or presence of KCl (24 mM), forskolin (FORS; 1 μM), or mastoparan (MP; 30 μM). *N* = 3 to 6; *n* = 12 to 75. Mann-Whitney (Benjamini, Krieger, and Yekutieli); *2*P* < 0.05, **2*P* < 0.01 and ****2*P* < 0.0001.

Subsequently, we measured a distal outcome of transmembrane ion fluxes, that is, insulin secretion from islets (Fig. 4). At low glucose levels (G3), no difference was observed between WT and GluDTR islets. G8 stimulated secretion from WT islets about fivefold but only 2.3-fold in GluDTR islets, and the addition of AAM increased secretion in WT islets 10-fold versus only threefold in GluDTR islets (Fig. 4A). The addition of glucagon largely restored insulin secretion in GluDTR islets to WT levels regardless of the absence or presence of AAM. Similarly, GLP-1 increased glucose-induced insulin secretion in WT and GluDTR islets in the absence or presence of AAM. However, in contrast to glucagon, GLP-1 restored secretion in GluDTR only to about 70% of levels observed in WT islets (Fig. 4A). We also examined the effect of molecules that act downstream from receptors (Fig. 4B). Forskolin (1 μM), a direct activator of adenylyl cyclases, induced a robust enhancement of insulin secretion at G8.2 in WT islets in the absence or presence of AAM, whereas only a far smaller change was observed in GluDTR islets (17.0 ± 4.3% of WT at G8.2 and 46.6 ± 10.2 of WT at G8.2 with AAM). Increasing the forskolin concentration to 10 μM at G8.2, the addition of the phosphodiesterase inhibitor 3-isobutyl-1-methylxanthine (IBMX; 0.1 mM) to forskolin at G8.2 or the use of forskolin at G16.7 stimulated GluDTR islets to a far lesser degree than WT islets (fig. S2). Likewise, membrane depolarization by potassium chloride (KCl; at G3) produced a considerable increase in insulin secretion but only in WT islets. Last, mastoparan, a direct stimulator of exocytosis (*37*), increased insulin secretion to a similar extent in WT and GluDTR islets. Collectively, these data indicate a major role for glucagon as well as for cAMP-dependent and cAMP-independent pathways in the observed phenotypes.

### α cells enhance β cell synchrony and cluster size

**Fig. 5.**
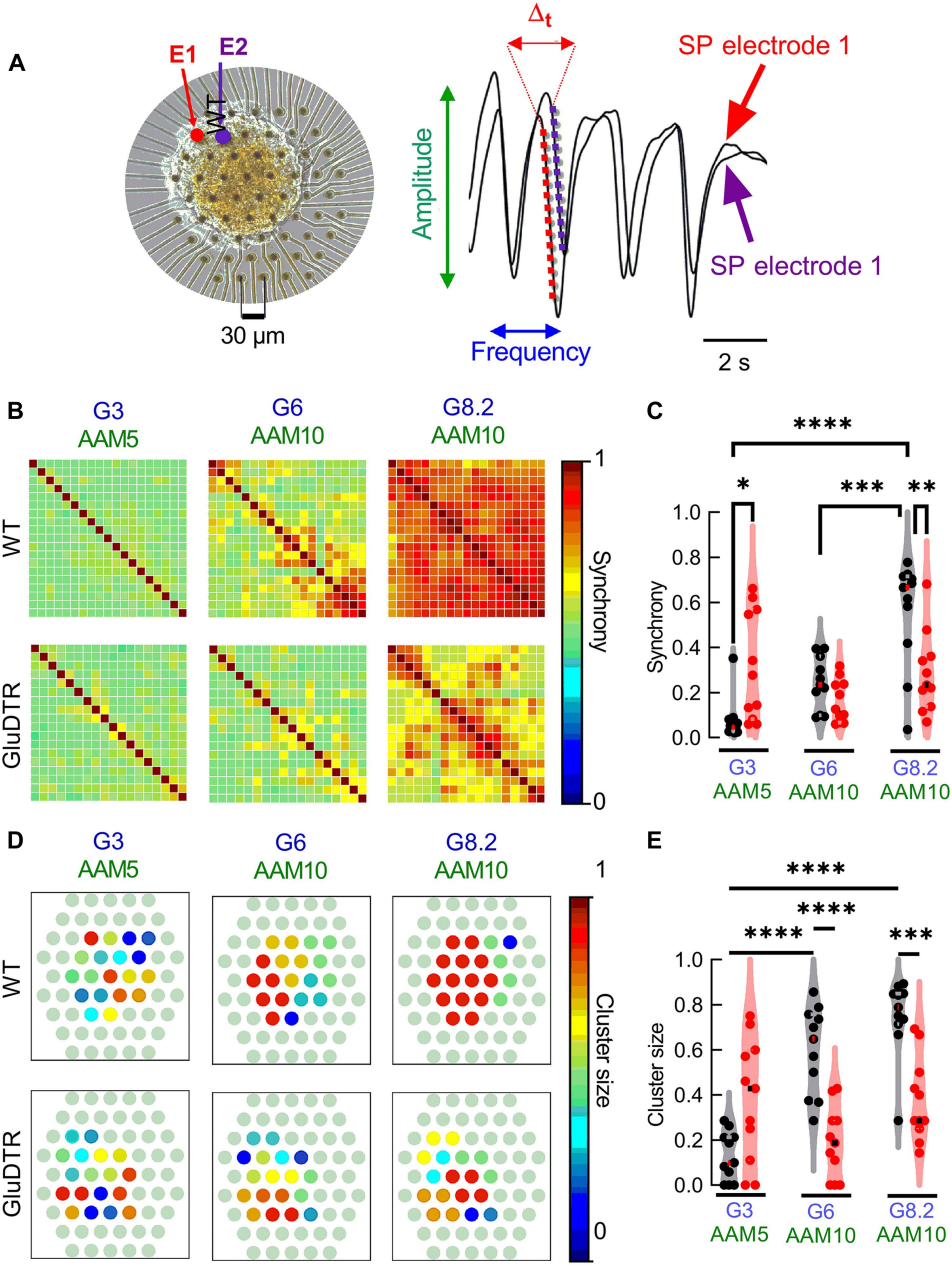
Effect of glucose and amino acids on synchrony and clusters in WT and GluDTR islets. (**A**) Image of an islet on an HD-MEA and example of two traces obtained from two neighboring electrodes. The electrodes (Ø 10 μm) are spaced 30 μm apart (electrode borders). (**B**) Correlation plot, i.e., synchrony within islets exposed to different concentrations of glucose (G3, G6, and G8.2) and amino acids (AA5 and AA10). Each tile represents an electrode pair. (**C**) Statistics of (B). (**D**) R spatial representation of cluster size. Representative outputs of cluster sizes (not means) in the different conditions are given. The normalized size of different clusters is indicated by color codes (normalization to the size of each islet). (**E**) Statistics on cluster sizes. *N* = 3 (animals); *n* = 11 islets for each condition. Two-way ANOVA and Tukey; *2*P* < 0.05, **2*P* < 0.01, ***2*P* < 0.001, and ****2*P* < 0.0001.

Next, we set out to decipher the dynamic modulation of the intraislet β cell networks by the presence/absence of α cells during physiological stimulation by glucose and amino acids. To this end, we used MEAs with high-density coverage by electrodes (HD-MEAs) to monitor regional changes in electrical activity within islets. As shown in Fig. 5A, electrodes are spaced 30 μm apart corresponding to about two to three β cell diameters. As extracellular recordings represent the inverse of intracellular recordings in terms of the direction of changes in potentials, the falling phase of the SPs corresponds to the synchronized depolarizations of β cells near the electrode (*31*). SPs can be correlated with establishing correlation matrices and the presence of clusters (>1 correlated electrode; Fig. 5A). Pairwise identifications of phase shifts allow us to establish the order of activation, leader regions, extent of connectivities within islets, and speed of propagation (fig. S3). Original recordings are given in fig. S4 and show again the large reduction of electrical activity in GluDTR islets as compared to WT islets in response to glucose and amino acids. Given that differences in electrical activity between WT and GluDTR islets were most marked in the presence of amino acids, we have concentrated our analysis on the effects of glucose in the presence of AAM. As shown in Fig. 5 (B and C), synchrony in WT islets increased considerably from G3/5 mM AAM to G6/10 mM AAM and G8.2/10 mM AAM. Synchrony in GluDTR islets at G3/5 mM AAM was widely scattered, but the cluster remained small and, at G8.2/10 mM AAM, a strong difference was evident between WT and GluDTR islets. Figure 5D gives an example of the distribution of synchronous electrodes and the presence of multiple clusters of different sizes. Increasing glucose from G3 to G6 and G8.2 with a concomitant increase in amino acids considerably increased the relative size of clusters in WT islets from 0.1 to 0.6 and 0.8, whereas in GluDTR islets, no significant change was observed. This was also apparent when grouping clusters according to their sizes and looking at their distribution (fig. S5). Whereas cluster sizes of WT islets increased already at G6/10 mM AAM, this was only apparent in GluDTR islets at G8.2/10 mM AAM. Moreover, GluDTR islets never formed very large clusters. Thus, cluster sizes at stimulatory glucose and amino acid concentrations were significantly smaller in GluDTR islets. To test which of the two SP qualities, frequency or amplitude, is most closely related to network properties, we also performed a correlation analysis of cluster sizes, synchrony, mean frequencies, and amplitudes at G3, G6, and G8.2 in the presence of AAM (fig. S6). Synchrony correlated significantly with SP amplitudes and confirms that amplitudes may serve as a surrogate for the extent of β cell coupling.

### α cells increase β cell cluster leader regions and signal speed but not their life span

**Fig. 6.**
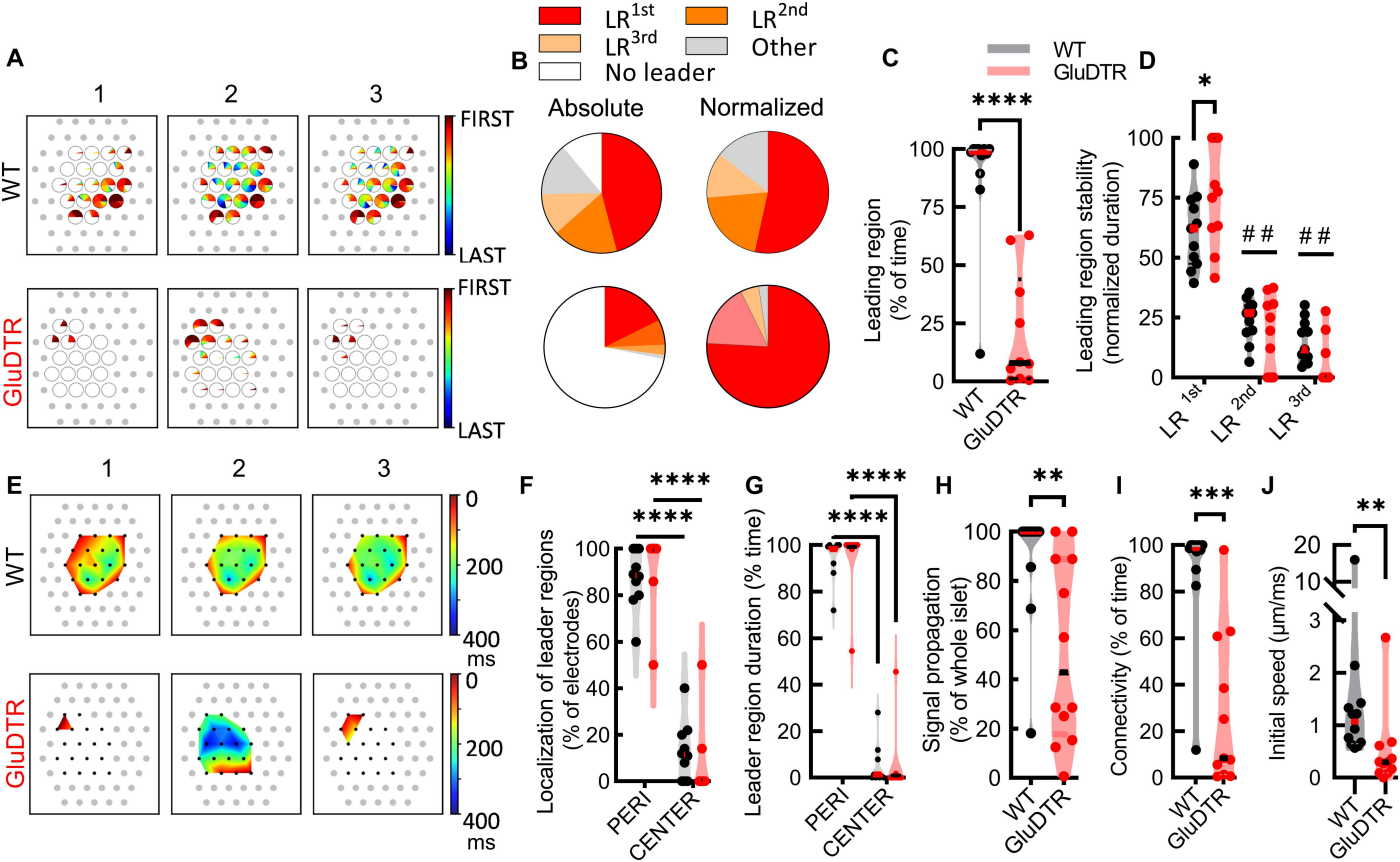
Properties of leader regions in WT and GluDTR islets during stimulation with G8.2 and amino acids. (**A**) Example for the appearance order of activity (first to last) and relative distribution of electrodes (“regions”) as pie charts under one islet (WT or GluDTR) for (1) the first 300 s of stimulation, (2) following 300 s, and (3) last 300 s. Open/filled circles, electrodes covered by an islet; gray dots, noncovered electrodes. (**B**) Absolute and normalized frequencies of the same electrode positions as leader region frequencies (first most often; second and third most often; others, fourth and more most often) for all experiments. The normalized pie chart excludes the absence of leader regions. (**C**) Statistics of leading regions in WT or GluDTR during the three periods. WT, black; GluDTR, red. (**D**) Appearance of the same electrode throughout phases 1, 2, and 3 as the most common leading region (LR^1st^) and second or third most common leading region (LR^2nd^ or LR^3rd^). (**E**) Example of propagation time of activation for one islet (WT or GluDTR); color-coded timescale from 0 to 400 ms (right *y* axis) during three periods [1, 2, and 3, as in (B)]. Black dots, electrodes covered by islets; gray dots, other electrodes. (**F**) Localization of leader regions in islet periphery (PERI) or center (CENTER). (**G**) Duration of the leader regions in the periphery or center of the islets. (**H**) Extent of spatial signal propagation within islets. (**I**) Duration of connectivity between regions for combined periods 1, 2, and 3, given as the percentage of time. (**J**) Initial speed of signal propagation (between the first leading electrode and the following electrode). *N* = 3 (animals); *n* = 11 islets for each condition. (C) and (F) to (H): Mann-Whitney; (D), (I), and (J): two-way ANOVA and Tukey; *2*P* < 0.05,**2*P* < 0.01, ***2*P* < 0.001, and ****2*P* < 0.0001; ^##^2*P* < 0.01 as compared to corresponding LR^1st^.

We subsequently addressed the spatial origin of electrical activity, which we termed “leader region,” and the spatial evolution of islet activation (Fig. 6). To that end, the 20-min stimulation period (G8.2/10 mM AAM) was divided in three consecutive periods, and the relative ranking of a given electrode as the leading region during that period was determined and presented with a color code in a pie chart. Figure 6A shows an example of a single WT or GluDTR islet. Only a limited number of electrodes were marked with red segments (indicating first leader regions) in most of their pie charts, although clearly distinct and widely separated regions could take the lead. While the number of active regions is fairly reduced in GluDTR islets, again, red segments were concentrated in a few regions. As there was little evidence for clear cut differences between the three time periods, we pooled them for a global analysis (Fig. 6, B to K). To indicate the relative frequency at which an electrode was present, the most prevalent was termed the first leading region (LR^1st^), and the second or third most prevalent leading region was termed LR^2nd^ or LR^3rd^, respectively. Also, given are regions that occur at an even lower frequency (“other”) as well as the absence of any detectable leading region. As there was a large difference between WT and GluDTR islets in regard to the presence of any leading region, we also normalized the data by excluding the occurrence of no discernible leading region (Fig. 6B).

In WT islets, the same region was the leading one for over half of the time, and during 85% of the time, the leading region is defined by just three different electrodes that may, however, be widely spaced apart. The situation is similar in GluDTR islets where, in two-thirds of the events, the connectivity started in the same region. To gain more insight on the characteristics of the leader regions, we performed statistics on the stability of the most frequent leading region as well as the second and third most often occurring regions (Fig. 6C). The life span of leading regions, i.e., stability, was significantly different in both types of islets between the most frequent leading regions and the less frequently occurring regions, LR^2nd^ and LR^3rd^ (Fig. 6D). Notably, the stability did not vary between WT and GluDTR: A given LR^1st^ lasted for about 20 to 30 s in WT as well as GluDTR islets, with maximal durations of more than 100 s, and decreased to about 10 s for LR3^rd^ (fig. S7, A and B). The mean distance between successive leader regions in WT islets was 88 ± 9 μm, which is almost three electrodes apart, and decreased significantly to 62 ± 7 μm in GluDTR islets (fig. S7C).

Next, we examined the propagation of the activation throughout the islets. As given in the examples here (Fig. 6E), activation in most instances started from the periphery in WT as well as in GluDTR islets, and peripheral leader regions were more stable than central ones (Fig. 6, F and G). In WT islets, this most often led to an activation throughout the entire islet (Fig. 6H), whereas a high variability was observed in GluDTR islets. Moreover, in WT islets, a network (termed connectivity) was maintained during most of the time, whereas this was the case for only about 10% of the time in GluDTR islets (Fig. 6I). The initial speed of activation, defined as the time lag between leading and following regions, was significantly slowed down from 2.3 ± 1.2 μm/s in WT islets to 0.5 ± 0.2 μm/s in GluDTR islets (Fig. 6J).

## DISCUSSION

The view on α cells has evolved from a mainly counterregulatory task in glucose homeostasis to more nuanced intraislet cooperativity in the handling of the physiological nutrients. However, a detailed view on ensuing β cell activity and networks is still lacking especially in electrophysiological terms. Our analysis of GluDTR mice confirms that α cells are pivotal for nutrient homeostasis including amino acids in vivo and islet activity in vitro in mice. Although the effect of amino acids on glucagon secretion was known for a long time, previously, in vivo experiments have mainly been conducted, with rare exceptions (*40*, *41*), using only one or some selected amino acids, which may not represent a physiological situation resulting from the wide differences in potency of individual amino acids on glucagon as well as insulin secretion (*37*).

The use of extracellular electrophysiology now defines precisely the α cell–mediated effect of amino acids on β cells. The activation of β cells in islets proceeds by two phases. Whereas the short-lasting first phase is characterized by a high activity with little coupling among β cells, the second phase exhibits a lower frequency of activity but a higher degree of coupling reflected by increased amplitudes and synchrony (*31*). Note that, in vitro, the first electrical or secretory phase is induced by a square pulse of nutrients and short-lasting, a few minutes, as compared to the insulin secretion peak in vivo, attained only after 10 to 15 min and dictated by a gradual increase in glucose concentrations. In WT islets, AAM increased the mean SP frequency in the first and second phases at G6 and G8.2. The mean amplitude changed only slightly with AAM at G8.2, likely because glucose alone already reached almost maximal stimulation (*34*). Notably, GluDTR islets consistently showed a lower mean frequency and amplitude of the second phase when amino acids were present. The amplitude of SPs varies with the degree of β cell coupling (*31*), and the reduced amplitudes observed are consistent with the observed smaller cluster size and lesser coupling in GluDTR islet β cells. In the absence of amino acids and the sole presence of glucose, we did not observe a significant difference in mean electrical activities between WT and GluDTR islets despite the fact that GluDTR islets secreted considerably less insulin under those conditions as also has been reported in other studies using the GluDTR model or β cell–specific knockout of the glucagon or GLP-1 receptor (*8*, *10*, *22*, *42*). The discrepancy between reduced insulin secretion in vitro and preserved insulin secretion and glucose tolerance in vivo (in the presence of glucose only) is probably due to increased GLP-1 levels in vivo in GluDTR mice (*22*). The difference in vitro between unaltered electrical activity and reduced secretion observed upon stimulation by glucose alone suggests that α cell hormones may alter signal transduction distal from ion fluxes in β cells. It is known that glucagon or GLP-1 receptors can activate not only adenylate cyclases but also phospholipases and phosphatidylinositol 3-kinase, which all modify insulin secretion independently from ion fluxes (*43*–*46*).

We were not able to restore in vitro electrical activity or secretion by forskolin, a general activator of adenylyl cyclases, as had been reported by others measuring secretion in the GluDTR model in the same genetic background (*22*) or in glucagon receptor–deficient mice islets (*8*). We have no ready explanation for the difference in forskolin effects but are intrigued by the fact that only forskolin but not the general phosphodiesterase inhibitor IBMX had been reported to restore secretion in glucagon receptor knockout mice (*8*). This points toward the requirement of extremely high levels of cAMP as low doses of IBMX already increase cAMP in β cells to a larger degree than glucose itself (*47*). The final steps in secretion were not changed between WT and GluDTR islets as mastoparan, a direct stimulator of exocytosis (*39*), caused comparable effects.

Our recovery experiments on electrical activity and insulin secretion suggest that glucagon is the major α cell–derived mediator. Notably, the differences in transmembrane ion fluxes and in insulin secretion observed in GluDTR islets were recovered by glucagon, as reported previously for hormone secretion (*22*). In contrast, GLP-1 was unable to fully restore insulin secretion when used at a concentration at which it had been reported to restore secretion in GluDTR mice (*22*), although the GLP-1 receptor antagonist exendin 9 reduced activity in WT islets stimulated with glucose and amino acids. Eventually, both receptors, those for GLP-1 and for glucagon, have to be activated to recover WT-like activity as glucagon is known to act via these two receptors (*10*, *16*, *48*). Collectively, this pinpoints to the pivotal role of glucagon acting via both glucagon and GLP-1 receptors, as suggested by another study (*11*).

The use of MEAs and HD-MEAs has advantages and limitations. Electrophysiology offers a far greater time resolution than the optical means where the frequency of data acquisition is generally limited to about 0.5 or 1 Hz for a few cell layers as compared to the kilohertz range in our case. Note that most recent work was capable of resolving events such as action potentials for a short time span at kilohertz line scans (*49*) or imaging entire islets at 2 Hz with a single-cell resolution (*50*). In contrast to imaging, even HD-MEAs lack a single-cell resolution, and we can only determine regions. Moreover, SPs represent summation signals from more than one single cell (*31*). This precludes determination of single-cell origin, but their analysis considerably reduces noise and increases robustness. Moreover, the multicellular origin also provides the extracellular electrodes at the islet surface with a penetration depth that may encompass the entire islet (*31*). Note that most of the imaging leading to the concept of hubs and leaders was done by recording only one or two layers of the islet surface because of technical constraints by acquisition speed and probe penetrance (*51*, *52*). Most recently, a very elegant approach has been reported using multiple MEA meshes with a lower resolution (electrodes 60 μm apart and electrode center to center) but which permit recording from intraislet sites (*53*). However, this powerful approach requires islet dissociation and reaggregation into pseudoislets, a procedure that may change native topology.

In contradistinction to ion-sensitive imaging, MEA recordings measure the sum of all ion species. Although, quantitatively, a substantial part of the observed changes in field potentials is carried by Ca^2+^-mediated depolarization in β cells (*27*), calcium imaging measures relative cytosolic calcium levels, whereas extracellular electrophysiology measures the outcome of the full ensemble of ionic fluxes, including repolarizing ions (K^+^). Moreover, signals from calcium imaging result from a combination of calcium entry, intracellular release, buffering and extrusion, and their kinetics. This may explain the notion that SPs are faster phenomena occurring at a higher frequency than the Ca^2+^ hub or wave initiator/leader cell activities or Ca^2+^ waves that have been used for network analysis by imaging. Whereas SPs last around 1 s (*31*, *54*), reported durations of wave initiator/leader or hub activities are in the range of 5 s and more, and waves last several minutes (*51*, *52*, *55*, *56*).

Another limitation of our study is the absence of vascularization especially as the intraislet blood flow is often thought to proceed vectorially from β to α cells, and thus, in native settings, β cells would receive little input from α cells, especially in the rodent islet. This issue is a matter of discussion as different vascular perfusion patterns have been described and α cells are not only present in the mantle of rodent islets (*57*, *58*).

Islet β cells are functionally heterogeneous (*59*, *60*) and achieve physiological effects by coupling and networks driven by gap junctions and cell-intrinsic dynamics (*56*, *61*). Initial work based on calcium imaging has pointed to the presence of a stable number of few hub or leader cells with distinct expression profiles, which initiate wave-like islet activity (*51*, *52*, *61*), although a more dynamic and nuanced behavior of leaders has been reported more recently (*50*, *62*–*64*). The spatial organization of electrical activity in WT islets indicated a preponderance of a defined region as the starting point for cluster formation. However, despite this marked occurrence in a given region, the leading region was not at all limited to one area, but intermittently, other distant regions of the islet took the lead. Even though our data contain only a limited spatial resolution, these observations are not easily reconcilable with the presence of defined and stable leaders at least under our more physiological conditions, i.e., the stimulation by glucose and amino acids. Signals were propagated about one order of magnitude faster in our MEA work as compared to calcium waves measured by imaging. However, we determined the initial velocity and not the mean speed, which slows down across the islet because of the resistive-capacitive properties of β cells (*65*, *66*). Moreover, all our MEA work has been performed at physiological extracellular calcium (1.2 mM). Raising external calcium to 2 mM or more, as most often used in imaging, increases the signal amplitude as well as duration and considerably slows down the frequencies (*31*). Last, the decreased velocity observed in GluDTR islets indicates that α cells increase the velocity and α cells are strongly stimulated by amino acids. Note also that previous values of velocity were obtained only upon glucose stimulation but not in the presence of amino acids and may be underestimated.

SP activity often started at the border of the islets, similar to what has been reported for Ca^2+^ waves in slices or whole-islet imaging (*50*, *67*, *68*). In rodent islets, α cells are more often present in the periphery, and preproglucagon-derived peptides may lower the activation threshold. However, we do not think that this predilection for the periphery may be brought upon by the special α/β cell topography as this signal topology was also apparent here in GluDTR islets, which renders this mechanism unlikely. Coupling among β cells does not only provide an efficient means for concerted activation but will also increase the activation threshold, thus dampening β cell activity (*69*–*71*). As β cells have fewer neighboring cells for coupling at the periphery of the islets, their activation threshold may be lower.

The analysis of synchrony and cluster size revealed considerable smaller clusters in GluDTR islets and an overall less organized functional activity at increased glucose and amino acids, which is reflected also in diminished insulin secretion. Proglucagon-derived peptides are known to lower the glucose-dependent activation threshold of islet β cells (*16*, *72*, *73*), and their absence may explain the lower fraction of active islet β cells in GluDTR islets as well as reduced initial speed and signal propagation. Notably, the life span of first, second, and third leader regions (LR^1st^, LR^2nd^, and LR^3rd^, respectively) was not different in GluDTR. Thus, the frequency of activation, speed, and coupling is regulated by α cells. In GluDTR islets, probably only those leader regions with the lowest activation threshold respond, and the presence of α cell hormones, such as in WT islets, lowers the general activation threshold and increases the total number of leader regions, propagation speed, and extension.

In mice and men, a close proximity as well as functional interaction between islet α and δ cells has been reported with repercussions on β cell activity (*74*, *75*). One may expect that the loss of α cells in GluDTR islets may reduce the activity of δ cells and consecutively relieve their inhibitory input on β cells. We have, however, only observed a strong reduction of insulin secretion and electrical activity upon stimulation with glucose and amino acids, a regime that also strongly stimulates the δ cells (*76*). As we have not addressed specifically here the interaction between δ and β cells, remodeling of more discrete effects such as concentration-dependent glucose sensitivity or counterregulations (*74*) cannot be excluded in GluDTR islets.

Insulin secretion in vivo upon glucose and amino acid tolerance tests was reduced during the first 30 min in GluDTR mice and only normalized afterward, in contrast to in vitro insulin secretion. This reduced initial insulin secretion in vivo provides strong evidence that α cells are not only required upon metabolic stress as thought previously (*8*, *19*–*21*) but also under more physiological conditions. This specific observation combined with electrophysiological characterization allows us to propose that the α-β interaction via proglucagon peptides is required for proper islet activity to face glucose and amino acids as nutrients via the functional properties of the β cell network. Elegant in-depth characterization of mice harboring only β cells has demonstrated that these animals have improved glucose tolerance and a highly adequate β cell function even under metabolic stress (*23*). It will be interesting to test whether more equilibrated exposure to nutrients, including also amino acids, may reveal differences and potentially altered functional β cell networks in this model. Detailed knowledge of the dynamic role of α cells may also help to improve currently used algorithms that control therapeutic insulin delivery in diabetes in man and are mainly based on glucose sensing (*77*, *78*).

## MATERIALS AND METHODS

### Materials

Glucagon, forskolin, and mastoparan were purchased from Sigma-Aldrich (St. Louis, MO), and exendin 9-39 and GLP-1 were purchased from Bachem (Bubendorf, Switzerland).

### Animals and islets

GluDTR and corresponding WT mice (in the C57BL/6N genetic background) were provided by M. Donath (*19*, *22*). The glucagon-DTR transgenic mice were generated by the Herrera group via pronuclear injection using heparin-binding epidermal growth factor–like growth factor [the natural receptor to DT (DTR)] cDNA subcloned downstream of a 1.6-kb-long rat glucagon promoter fragment (*19*, *79*). The expression of DTR is α cell–specific (*22*, *80*) and does not alter islet morphology beyond α cell ablation (*19*). DT [D0564; Sigma-Aldrich; dissolved in 0.9% (w/v) NaCl] was injected in 5- to 6-week-old WT and GluDTR animals in three intraperitoneal injections of 500 ng each on days 1, 3, and 5, as reported before (*19*, *22*). Mice were used for experiments 6 to 20 weeks after injections. For genotyping, the following primers were used: G1-52S: 5′-GAG AAA TTT ATA TTG TCA GCG-3′; DTR reverse 4: 5′-CTT CAG CAC CAC CGA CGG C-3′, resulting in a ~0.8-kb polymerase chain reaction (PCR) transcript. PCR was performed as published by Traub *et al.* (*22*). Mice were euthanized by cervical dislocation according to University of Bordeaux ethics committee guidelines (authorization no. 2087-2019052917497896). Islets were obtained by enzymatic digestion and handpicking (*29*, *31*, *32*).

### Study approval

All animal experiments were reviewed by the Bordeaux University Ethical Committee with the corresponding government authorization (PA no. 2087-2019052917497896).

### In vivo characterizations

Glucose (2 g or 3/kg body weight) and amino acids (1 g/kg body weight) or pyruvate (2 g/kg body weight) were administered by intraperitoneal injection after 6 hours of fasting (8:00 to 14:00) (*32*). Blood samples were obtained by tail tip bleeding and assayed using a glucometer (Freestyle, Abbott) and collected on a hematocrit capillary tube (Hirschmann 9100275). Blood samples collected at different time points were transferred to low-binding Eppendorf tubes (Sorenson 11300) and centrifuged for 15 min at 1800*g* at 4°C, and the plasma was transferred in a new tube and frozen at 20°C

### Insulin secretion

Insulin secretion was determined under static conditions. After 4 days of culture in RPMI medium, to allow recovery from isolation-induced stress and in line with the duration of islet culture on MEAs, 40 islets were placed on small filters inserted into the wells of a 48-well plate (PluriStrainer Mini 40 μm, Dutscher) containing 500 μl of EPHYS solution (for the composition, see the “Electrophysiology” section below). Experiments were conducted in this buffer to ensure comparability with MEA experiments. The filters allowed easy and rapid transfer of islets from one well to another. All incubations were performed at 37°C and 5% CO_2_ for 30 min. An initial incubation served to wash the islets from the culture medium, followed by basal (G3) and stimulated conditions. For stimulations in the presence of amino acids, the same islets and wells were kept for 3 hours in culture medium before washes and incubations as above. Last, the islets were placed in ethanolic acid at −20°C for at least 12 hours. All solutions were collected and centrifuged at 800*g* for 5 min, and 300 μl of the supernatant was stored at −20°C. Islets were taken up in acid-ethanol and kept at −80°C until analysis. Insulin concentrations were determined by enzyme-linked immunosorbent assay (Mercodia mouse insulin; 10-1247-01) as was glucagon (Mercodia Glucagon 10-1281-01).

### RNA extraction and quantitative PCR

Total RNA of isolated mouse islets was extracted using the NucleoSpin RNA II Kit (Macherey Nagel). cDNA was prepared with random hexamers and Superscript II reverse transcriptase (Invitrogen). For quantitative PCR, the real-time PCR system 7500 (Applied Biosystems) with BRYT Green Dye (Promega) and the following primers was used: Gcg: 5′-TTACTTTGTGGCTGGATTGCTT-3′ and 5′-AGTGGCGTTTGTCTTCATTCA-3′; 18S: 5′-GGGAGCCTGAGAAACGGC-3′ and 5′-GGGTCGGGAGTGGGTAATTT-3′. Gene expression was analyzed with the comparative 2^ΔΔCT^ method.

### Electrophysiology

Islets were seeded on MEAs coated with Matrigel (2%, v/v) (BD Biosciences, San Diego, CA) and cultured at 37°C (5% CO_2_ and 0.9% relative humidity) in RPMI medium (11 mM glucose; Thermo Fisher Scientific, Waltham, MA), and medium was changed every other day as described (*31*, *32*, *36*). Experiments were performed at 37°C in EPHYS buffer containing 135 mM NaCl, 4.8 mM KCl, 1.2 mM MgCl_2_, 1.2 mM CaCl_2_, 10 mM Hepes, and glucose and amino acids as indicated (pH 7.4 adjusted with NaOH). The 10 mM AAM (AAM10) was according to Zhu *et al.* (*24*) and contained the following: 0.88 mM Ala, 0.38 mM Arg, 0.076 mM Asp, 0.19 mM Cit, 0.24 mM Glut, 0.6 mM Gly, 0.15 mM His, 0.19 mM Ile, 0.32 mM Leu, 0.74 mM Lys, 0.1 mM Met, 1.4 mM Orn, 0.16 mM Phe, 0.7 mM Pro, 1.14 mM Ser, 0.54 mM Thr, 0.15 mM Trp, 0.4 mM Val, and 2 mM Glut. AAM5 contained half the amount of each amino acid. Extracellular recordings were performed on MEA placed in a MEA recording system {USB-MEA60-Inv-System-E amplifier [Multi-Channel Systems GmbH (MCS)]; gain: 1200; MCS, Reutlingen, Germany} controlled by MC_Rack software (version 4.6.2; MCS). Recordings of different intraislet regions were performed using HD-MEAs (60HexaMEA40/10iR-ITO-gr, 59 TiN electrodes, Ø 10 mm, 30 mm between electrodes, border to border) that were continuously perfused at 0.5 ml/min (Reglo ICC; Ismatec, Glattbrugg, Switzerland). Extracellular field potentials were acquired at 10 kHz, amplified, and bandpass filtered at 0.1 to 3000 Hz (*29*–*31*, *34*–*36*). Images of islets on MEAs were taken before and after each experiment to localize electrodes covered with islets. Electrophysiological data were analyzed with MC_Rack software. SPs were isolated using a 0.1- to 2-Hz bandpass filter, and frequencies were determined using the threshold module of MC_Rack with a dead time (minimum time between two events) of 300 ms. The peak-to-peak amplitude module of MC_Rack was used to determine SP amplitudes.

### Analysis of HD-MEA recordings

Synchrony measurements, phase measurements, clustering, and order of activation were computed with custom Python software using libraries numpy, pandas, and scipy. Libraries matplotlib and seaborn were also used for data visualization. Electrophysiological data were filtered using 0.2- to 2.0-Hz Bessel filters and resampled at 100 Hz. Synchrony between signals was measured using pairwise Pearson correlation between signals from all electrodes (fig. S1). From the resulting correlation matrix, clusters of synchronized electrodes were identified through hierarchical clustering using UPGMA (unweighted pair group method with arithmetic mean). The clustering threshold was set to 70% of the maximum distance between signals as calculated in the linkage matrix. The lag between signals was measured by computing the cross-correlation between signal pairs and retrieving the lag at maximum correlation for each pair. Because of the 100-Hz sample rate, the time resolution for phase measurements was 10 ms. All lag values that yielded a maximum correlation below 0.7 were discarded. To monitor changes over time, the signal lag was computed in a 30-s sliding window, with steps of 7.5 s (75% overlap). The order of activation of electrodes was deduced from the windowed lag measurements by identifying the leader in each window (signal of the earliest lag) and sorting the others by ascending phase, relative to the leader. For periphery versus central analysis, electrodes were classified on the basis of their relative position with respect to the islet outline. The peripheral region was defined as the first continuous outer line of electrodes covering the islet boundary. All remaining electrodes located within this peripheral layer were classified as central. This procedure was repeated for each individual islet to account for variability in islet size and position.

### Statistics

Graphics, quantifications, and statistics were performed with Prism software (version 7; GraphPad, La Jolla, CA). Data are presented as the means and SEM. The minimal value of mean SP frequency after the first peak (corresponding to the nadir) was taken as the limit between phases. Gaussian distributions were tested by the Shapiro-Wilk test, and statistical tests were performed as indicated in the figure legends.

## References

[1] GBD 2021 Diabetes Collaborators, Global, regional, and national burden of diabetes from 1990 to 2021, with projections of prevalence to 2050: A systematic analysis for the Global Burden of Disease Study 2021. Lancet 402, 203–234 (2023). 37356446 10.1016/S0140-6736(23)01301-6 PMC10364581

[2] M. Bakhti, A. Bottcher, H. Lickert, Modelling the endocrine pancreas in health and disease. Nat. Rev. Endocrinol. 15, 155–171 (2019). 30504925 10.1038/s41574-018-0132-z

[3] S. L. Armour, J. E. Stanley, J. Cantley, E. D. Dean, J. G. Knudsen, Metabolic regulation of glucagon secretion. J. Endocrinol. 259, e230081 (2023). 37523232 10.1530/JOE-23-0081 PMC10681275

[4] T. S. Morriseau, C. A. Doucette, V. W. Dolinsky, More than meets the islet: Aligning nutrient and paracrine inputs with hormone secretion in health and disease. Am. J. Physiol. Endocrinol. Metab. 322, E446–E463 (2022). 35373587 10.1152/ajpendo.00411.2021

[5] B. A. Menge, L. Grüber, S. M. Jørgensen, C. F. Deacon, W. E. Schmidt, J. D. Veldhuis, J. J. Holst, J. J. Meier, Loss of inverse relationship between pulsatile insulin and glucagon secretion in patients with type 2 diabetes. Diabetes 60, 2160–2168 (2011). 21677283 10.2337/db11-0251 PMC3142077

[6] B. Hellman, A. Salehi, E. Gylfe, H. Dansk, E. Grapengiesser, Glucose generates coincident insulin and somatostatin pulses and antisynchronous glucagon pulses from human pancreatic islets. Endocrinology 150, 5334–5340 (2009). 19819962 10.1210/en.2009-0600

[7] B. Hellman, A. Salehi, E. Grapengiesser, E. Gylfe, Isolated mouse islets respond to glucose with an initial peak of glucagon release followed by pulses of insulin and somatostatin in antisynchrony with glucagon. Biochem. Biophys. Res. Commun. 417, 1219–1223 (2012). 22227186 10.1016/j.bbrc.2011.12.113

[8] M. E. Capozzi, B. Svendsen, S. E. Encisco, S. L. Lewandowski, M. D. Martin, H. Lin, J. L. Jaffe, R. W. Coch, J. M. Haldeman, P. E. MacDonald, M. J. Merrins, D. A. D’Alessio, J. E. Campbell, β cell tone is defined by proglucagon peptides through cAMP signaling. JCI Insight 4, e126742 (2019). 30720465 10.1172/jci.insight.126742 PMC6483521

[9] K. D. Galsgaard, M. Winther-Sorensen, C. Orskov, H. Kissow, S. S. Poulsen, H. Vilstrup, C. Prehn, J. Adamski, S. L. Jepsen, B. Hartmann, J. Hunt, M. J. Charron, J. Pedersen, N. J. Wewer Albrechtsen, J. J. Holst, Disruption of glucagon receptor signaling causes hyperaminoacidemia exposing a possible liver-alpha-cell axis. Am. J. Physiol. Endocrinol. Metab. 314, E93–E103 (2018). 28978545 10.1152/ajpendo.00198.2017 PMC6048389

[10] B. Svendsen, O. Larsen, M. B. N. Gabe, C. B. Christiansen, M. M. Rosenkilde, D. J. Drucker, J. J. Holst, Insulin secretion depends on intra-islet glucagon signaling. Cell Rep. 25, 1127–1134.e2 (2018). 30380405 10.1016/j.celrep.2018.10.018

[11] C. Cui, D. C. Leander, S. M. Gray, K. El, A. Chen, P. A. Grimsrud, J. O. Becker, A. Taylor, G. F. Zhang, K. W. Sloop, C. B. Verchere, A. N. Hoofnagle, D. A. D'Alessio, J. E. Campbell, α cells use both PC1/3 and PC2 to process proglucagon peptides and control insulin secretion. Sci. Adv. 11, eady8048 (2025). 40971442 10.1126/sciadv.ady8048 PMC12448133

[12] K. Moens, H. Heimberg, D. Flamez, P. Huypens, E. Quartier, Z. Ling, D. Pipeleers, S. Gremlich, B. Thorens, F. Schuit, Expression and functional activity of glucagon, glucagon-like peptide I, and glucose-dependent insulinotropic peptide receptors in rat pancreatic islet cells. Diabetes 45, 257–261 (1996). 8549871 10.2337/diab.45.2.257

[13] R. L. Rodgers, Glucagon, cyclic AMP, and hepatic glucose mobilization: A half-century of uncertainty. Physiol. Rep. 10, e15263 (2022). 35569125 10.14814/phy2.15263 PMC9107925

[14] M. B. Wheeler, M. Lu, J. S. Dillon, X. H. Leng, C. Chen, A. E. Boyd III, Functional expression of the rat glucagon-like peptide-I receptor, evidence for coupling to both adenylyl cyclase and phospholipase-C. Endocrinology 133, 57–62 (1993). 8391428 10.1210/endo.133.1.8391428

[15] M. Shigeto, R. Ramracheya, A. I. Tarasov, C. Y. Cha, M. V. Chibalina, B. Hastoy, K. Philippaert, T. Reinbothe, N. Rorsman, A. Salehi, W. R. Sones, E. Vergari, C. Weston, J. Gorelik, M. Katsura, V. O. Nikolaev, R. Vennekens, M. Zaccolo, A. Galione, P. R. V. Johnson, K. Kaku, G. Ladds, P. Rorsman, GLP-1 stimulates insulin secretion by PKC-dependent TRPM4 and TRPM5 activation. J. Clin. Invest. 125, 4714–4728 (2015). 26571400 10.1172/JCI81975 PMC4665783

[16] O. Cabrera, J. Ficorilli, J. Shaw, F. Echeverri, F. Schwede, O. G. Chepurny, C. A. Leech, G. G. Holz, Intra-islet glucagon confers β-cell glucose competence for first-phase insulin secretion and favors GLP-1R stimulation by exogenous glucagon. J. Biol. Chem. 298, 101484 (2022). 34896391 10.1016/j.jbc.2021.101484 PMC8789663

[17] D. T. Hoang, M. Hara, J. Jo, Design principles of pancreatic islets: Glucose-dependent coordination of hormone pulses. PLOS ONE 11, e0152446 (2016). 27035570 10.1371/journal.pone.0152446 PMC4818077

[18] I. Garzilli, S. Itzkovitz, Design principles of the paradoxical feedback between pancreatic alpha and beta cells. Sci. Rep. 8, 10694 (2018). 30013127 10.1038/s41598-018-29084-4 PMC6048053

[19] F. Thorel, N. Damond, S. Chera, A. Wiederkehr, B. Thorens, P. Meda, C. B. Wollheim, P. L. Herrera, Normal glucagon signaling and beta-cell function after near-total alpha-cell ablation in adult mice. Diabetes 60, 2872–2882 (2011). 21926270 10.2337/db11-0876 PMC3198058

[20] J. Pedersen, R. K. Ugleholdt, S. M. Jørgensen, J. A. Windeløv, K. V. Grunddal, T. W. Schwartz, E. M. Füchtbauer, S. S. Poulsen, P. J. Holst, J. J. Holst, Glucose metabolism is altered after loss of L cells and α-cells but not influenced by loss of K cells. Am. J. Physiol. Endocrinol. Metab. 304, E60–E73 (2013). 23115082 10.1152/ajpendo.00547.2011

[21] C. Shiota, K. Prasadan, P. Guo, Y. El-Gohary, J. Wiersch, X. Xiao, F. Esni, G. K. Gittes, α-Cells are dispensable in postnatal morphogenesis and maturation of mouse pancreatic islets. Am. J. Physiol. Endocrinol. Metab. 305, E1030–E1040 (2013). 23982158 10.1152/ajpendo.00022.2013

[22] S. Traub, D. T. Meier, F. Schulze, E. Dror, T. M. Nordmann, N. Goetz, N. Koch, E. Dalmas, M. Stawiski, V. Makshana, F. Thorel, P. L. Herrera, M. Boni-Schnetzler, M. Y. Donath, Pancreatic α cell-derived glucagon-related peptides are required for β cell adaptation and glucose homeostasis. Cell Rep. 18, 3192–3203 (2017). 28355570 10.1016/j.celrep.2017.03.005

[23] M. Perez-Frances, E. Bru-Tari, C. Cohrs, M. V. Abate, L. van Gurp, K. Furuyama, S. Speier, F. Thorel, P. L. Herrera, Regulated and adaptive in vivo insulin secretion from islets only containing β-cells. Nat .Metab. 6, 1791–1806 (2024). 39169271 10.1038/s42255-024-01114-8 PMC11422169

[24] L. Zhu, D. Dattaroy, J. Pham, L. Wang, L. F. Barella, Y. Cui, K. J. Wilkins, B. L. Roth, U. Hochgeschwender, F. M. Matschinsky, K. H. Kaestner, N. M. Doliba, J. Wess, Intra-islet glucagon signaling is critical for maintaining glucose homeostasis. JCI Insight 5, e127994 (2019). 31012868 10.1172/jci.insight.127994 PMC6542600

[25] L. J. van Loon, W. H. Saris, H. Verhagen, A. J. Wagenmakers, Plasma insulin responses after ingestion of different amino acid or protein mixtures with carbohydrate. Am. J. Clin. Nutr. 72, 96–105 (2000). 10871567 10.1093/ajcn/72.1.96

[26] K. El, M. E. Capozzi, J. E. Campbell, Repositioning the alpha cell in postprandial metabolism. Endocrinology 161, bqaa169 (2020). 32964214 10.1210/endocr/bqaa169 PMC7899437

[27] P. Rorsman, F. M. Ashcroft, Pancreatic β-cell electrical activity and insulin secretion: Of mice and men. Physiol. Rev. 98, 117–214 (2018). 29212789 10.1152/physrev.00008.2017 PMC5866358

[28] J. Weitz, D. Menegaz, A. Caicedo, Deciphering the complex communication networks that orchestrate pancreatic islet function. Diabetes 70, 17–26 (2021). 33355306 10.2337/dbi19-0033 PMC7881851

[29] E. Puginier, K. Leal-Fischer, J. Gaitan, M. Lallouet, P. A. Scotti, M. Raoux, J. Lang, Extracellular electrophysiology on clonal human β-cell spheroids. Front. Endocrinol. 15, 1402880 (2024). 10.3389/fendo.2024.1402880 PMC11176477 38883608

[30] M. Jaffredo, N. A. J. Krentz, B. Champon, C. E. Duff, S. Nawaz, N. Beer, C. Honore, A. Clark, P. Rorsman, J. Lang, A. L. Gloyn, M. Raoux, B. Hastoy, Electrophysiological characterization of inducible pluripotent stem cell-derived human β-like cells and an SLC30A8 disease model. Diabetes 73, 1255–1265 (2024). 38985991 10.2337/db23-0776 PMC11262041

[31] M. Jaffredo, E. Bertin, A. Pirog, E. Puginier, J. Gaitan, S. Oucherif, F. Lebreton, D. Bosco, B. Catargi, D. Cattaert, S. Renaud, J. Lang, M. Raoux, Dynamic uni- and multicellular patterns encode biphasic activity in pancreatic islets. Diabetes 70, 878–888 (2021). 33468514 10.2337/db20-0214

[32] M. Abarkan, J. Gaitan, F. Lebreton, R. Perrier, M. Jaffredo, C. Mulle, C. Magnan, M. Raoux, J. Lang, The glutamate receptor GluK2 contributes to the regulation of glucose homeostasis and its deterioration during aging. Mol. Metab. 30, 152–160 (2019). 31767166 10.1016/j.molmet.2019.09.011 PMC6807305

[33] R. Perrier, A. Pirog, M. Jaffredo, J. Gaitan, B. Catargi, S. Renaud, M. Raoux, J. Lang, Bioelectronic organ-based sensor for microfluidic real-time analysis of the demand in insulin. Biosens. Bioelectron. 117, 253–259 (2018). 29909196 10.1016/j.bios.2018.06.015

[34] F. Lebreton, A. Pirog, I. Belouah, D. Bosco, T. Berney, P. Meda, Y. Bornat, B. Catargi, S. Renaud, M. Raoux, J. Lang, Slow potentials encode intercellular coupling and insulin demand in pancreatic beta cells. Diabetologia 58, 1291–1299 (2015). 25788295 10.1007/s00125-015-3558-z

[35] E. Puginier, A. Pirog, F. P. de Gannes, J. Gaitan, A. Hurtier, D. Chapeau, M. Monchablon, M. Raoux, S. Renaud, J. Lang, A micro-organ based microfluidic biosensor for continuous monitoring of glucose levels in vivo. NPJ Biosens. 3, 12 (2026).

[36] M. Lallouet, L. Olcomendy, J. Gaitan, K. Montiege, M. Monchablon, A. Pirog, D. Chapeau, E. Puginier, S. Renaud, M. Raoux, J. Lang, A microfluidic twin islets-on-chip device for on-line electrophysiological monitoring. Lab Chip 25, 1831–1841 (2025). 40042033 10.1039/d4lc00967c

[37] D. M. Rocha, G. R. Faloona, R. H. Unger, Glucagon-stimulating activity of 20 amino acids in dogs. J. Clin. Invest. 51, 2346–2351 (1972). 4639019 10.1172/JCI107046 PMC292401

[38] C. C. Hughey, D. H. Wasserman, R. S. Lee-Young, L. Lantier, Approach to assessing determinants of glucose homeostasis in the conscious mouse. Mamm. Genome 25, 522–538 (2014). 25074441 10.1007/s00335-014-9533-z PMC4167555

[39] S. G. Straub, R. F. James, M. J. Dunne, G. W. Sharp, Glucose augmentation of mastoparan-stimulated insulin secretion in rat and human pancreatic islets. Diabetes 47, 1053–1057 (1998). 9648828 10.2337/diabetes.47.7.1053

[40] K. Maruszczak, C. Rasmussen, F. R. Ceutz, A. Orgaard, E. Elmelund, M. M. Richter, J. J. Holst, M. Winther-Sorensen, N. J. Wewer Albrechtsen, Arginine-induced glucagon secretion and glucagon-induced enhancement of amino acid catabolism are not influenced by ambient glucose levels in mice. Am. J. Physiol. Endocrinol. Metab. 323, E207–E214 (2022). 35830690 10.1152/ajpendo.00122.2022

[41] M. Winther-Sørensen, K. D. Galsgaard, A. Santos, S. A. J. Trammell, K. Sulek, R. E. Kuhre, J. Pedersen, D. B. Andersen, A. S. Hassing, M. Dall, J. T. Treebak, M. P. Gillum, S. S. Torekov, J. A. Windeløv, J. E. Hunt, S. A. S. Kjeldsen, S. L. Jepsen, C. G. Vasilopoulou, F. K. Knop, C. Ørskov, M. P. Werge, H. C. Bisgaard, P. L. Eriksen, H. Vilstrup, L. L. Gluud, J. J. Holst, N. J. Wewer Albrechtsen, Glucagon acutely regulates hepatic amino acid catabolism and the effect may be disturbed by steatosis. Mol. Metab. 42, 101080 (2020). 32937194 10.1016/j.molmet.2020.101080 PMC7560169

[42] K. Suba, Y. Patel, A. Martin-Alonso, B. Hansen, X. Xu, A. Roberts, M. Norton, P. Chung, J. Shrewsbury, R. Kwok, V. Kalogianni, S. Chen, X. Liu, K. Kalyviotis, G. A. Rutter, B. Jones, J. Minnion, B. M. Owen, P. Pantazis, W. Distaso, D. J. Drucker, T. M. Tan, S. R. Bloom, K. G. Murphy, V. Salem, Intra-islet glucagon signalling regulates beta-cell connectivity, first-phase insulin secretion and glucose homoeostasis. Mol. Metab. 85, 101947 (2024). 38677509 10.1016/j.molmet.2024.101947 PMC11177084

[43] B. Roger, J. Papin, P. Vacher, M. Raoux, A. Mulot, M. Dubois, J. Kerr-Conte, B. H. Voy, F. Pattou, G. Charpentier, J. C. Jonas, N. Moustaid-Moussa, J. Lang, Adenylyl cyclase 8 is central to glucagon-like peptide 1 signalling and effects of chronically elevated glucose in rat and human pancreatic beta cells. Diabetologia 54, 390–402 (2011). 21046358 10.1007/s00125-010-1955-x

[44] K. Kaneko, K. Ueki, N. Takahashi, S. Hashimoto, M. Okamoto, M. Awazawa, Y. Okazaki, M. Ohsugi, K. Inabe, T. Umehara, M. Yoshida, M. Kakei, T. Kitamura, J. Luo, R. N. Kulkarni, C. R. Kahn, H. Kasai, L. C. Cantley, T. Kadowaki, Class IA phosphatidylinositol 3-kinase in pancreatic β cells controls insulin secretion by multiple mechanisms. Cell Metab. 12, 619–632 (2010). 21109194 10.1016/j.cmet.2010.11.005 PMC3736578

[45] Y. Suzuki, H. Zhang, N. Saito, I. Kojima, T. Urano, H. Mogami, Glucagon-like peptide 1 activates protein kinase C through Ca^2+^-dependent activation of phospholipase C in insulin-secreting cells. J. Biol. Chem. 281, 28499–28507 (2006). 16870611 10.1074/jbc.M604291200

[46] N. Zaïmia, J. Obeid, A. Varrault, J. Sabatier, C. Broca, P. Gilon, S. Costes, G. Bertrand, M. A. Ravier, GLP-1 and GIP receptors signal through distinct β-arrestin 2-dependent pathways to regulate pancreatic β cell function. Cell Rep. 42, 113326 (2023). 37897727 10.1016/j.celrep.2023.113326

[47] O. Dyachok, O. Idevall-Hagren, J. Sagetorp, G. Tian, A. Wuttke, C. Arrieumerlou, G. Akusjarvi, E. Gylfe, A. Tengholm, Glucose-induced cyclic AMP oscillations regulate pulsatile insulin secretion. Cell Metab. 8, 26–37 (2008). 18590690 10.1016/j.cmet.2008.06.003

[48] P. Huypens, Z. Ling, D. Pipeleers, F. Schuit, Glucagon receptors on human islet cells contribute to glucose competence of insulin release. Diabetologia 43, 1012–1019 (2000). 10990079 10.1007/s001250051484

[49] J. Dolenšek, V. Pohorec, M. Skelin Klemen, M. Gosak, A. Stožer, Ultrafast multicellular calcium imaging of calcium spikes in mouse beta cells in tissue slices. Acta Physiol. 241, e14261 (2025). 10.1111/apha.14261 PMC11726428 39803792

[50] E. Jin, J. K. Briggs, R. K. P. Benninger, M. J. Merrins, Glucokinase activity controls peripherally located subpopulations of β-cells that lead islet Ca^2+^ oscillations. eLife 13, RP103068 (2025). 39936635 10.7554/eLife.103068 PMC11820133

[51] N. R. Johnston, R. K. Mitchell, E. Haythorne, M. P. Pessoa, F. Semplici, J. Ferrer, L. Piemonti, P. Marchetti, M. Bugliani, D. Bosco, E. Berishvili, P. Duncanson, M. Watkinson, J. Broichhagen, D. Trauner, G. A. Rutter, D. J. Hodson, Beta cell hubs dictate pancreatic islet responses to glucose. Cell Metab. 24, 389–401 (2016). 27452146 10.1016/j.cmet.2016.06.020 PMC5031557

[52] V. Salem, L. D. Silva, K. Suba, E. Georgiadou, S. N. M. Gharavy, N. Akhtar, A. Martin-Alonso, D. C. A. Gaboriau, S. M. Rothery, T. Stylianides, G. Carrat, T. J. Pullen, S. P. Singh, D. J. Hodson, I. Leclerc, A. M. J. Shapiro, P. Marchetti, L. J. B. Briant, W. Distaso, N. Ninov, G. A. Rutter, Leader β-cells coordinate Ca^2+^ dynamics across pancreatic islets in vivo. Nat. Metab. 1, 615–629 (2019). 32694805 10.1038/s42255-019-0075-2 PMC7617060

[53] Q. Li, R. Liu, Z. Lin, X. Zhang, W. Wang, I. M. Galicia-Silva, M. Liu, Z. Gao, S. D. Pollock, J. R. Alvarez-Dominguez, J. Liu, Implanted flexible electronics reveal principles of human islet cell electrical maturation. Science 391, eaeb3295 (2026). 41712726 10.1126/science.aeb3295

[54] M. Abarkan, A. Pirog, D. Mafilaza, G. Pathak, G. N'Kaoua, E. Puginier, R. O'Connor, M. Raoux, M. J. Donahue, S. Renaud, J. Lang, Vertical organic electrochemical transistors and electronics for low amplitude micro-organ signals. Adv. Sci. 9, e2105211 (2022). 10.1002/advs.202105211 PMC8922095 35064774

[55] C. L. Lei, J. A. Kellard, M. Hara, J. D. Johnson, B. Rodriguez, L. J. B. Briant, Beta-cell hubs maintain Ca^2+^ oscillations in human and mouse islet simulations. Islets 10, 151–167 (2018). 30142036 10.1080/19382014.2018.1493316 PMC6113907

[56] J. K. Briggs, A. Gresch, I. Marinelli, J. M. Dwulet, D. J. Albers, V. Kravets, R. K. P. Benninger, β-Cell intrinsic dynamics rather than gap junction structure dictates subpopulations in the islet functional network. eLife 12, e83147 (2023). 38018905 10.7554/eLife.83147 PMC10803032

[57] A. Caicedo, M. O. Huising, J. Wess, An intraislet paracrine signaling pathway that enables glucagon to stimulate pancreatic β-cells. Diabetes 72, 1748–1750 (2023). 37983525 10.2337/dbi23-0023 PMC10658067

[58] G. C. Weir, S. Bonner-Weir, Conflicting views about interactions between pancreatic α-cells and β-cells. Diabetes 72, 1741–1747 (2023). 37983524 10.2337/db23-0292 PMC10658062

[59] R. K. P. Benninger, V. Kravets, The physiological role of β-cell heterogeneity in pancreatic islet function. Nat. Rev. Endocrinol. 18, 9–22 (2022). 34667280 10.1038/s41574-021-00568-0 PMC8915749

[60] G. A. Rutter, A. Gresch, L. Delgadillo Silva, R. K. P. Benninger, Exploring pancreatic beta-cell subgroups and their connectivity. Nat. Metab. 6, 2039–2053 (2024). 39117960 10.1038/s42255-024-01097-6 PMC13552999

[61] A. Stozer, M. Gosak, J. Dolensek, M. Perc, M. Marhl, M. S. Rupnik, D. Korosak, Functional connectivity in islets of Langerhans from mouse pancreas tissue slices. PLOS Comput. Biol. 9, e1002923 (2013). 23468610 10.1371/journal.pcbi.1002923 PMC3585390

[62] M. Duh, M. Šterk, L. Križančić Bombek, P. E. MacDonald, A. Stožer, M. Gosak, Spatially bound functional heterogeneity drives modular organization in beta-cell networks. Biophys. J. 124, 3008–3022 (2025). 40765144 10.1016/j.bpj.2025.07.043 PMC12709238

[63] V. Kravets, J. M. Dwulet, W. E. Schleicher, D. J. Hodson, A. M. Davis, L. Pyle, R. A. Piscopio, M. Sticco-Ivins, R. K. P. Benninger, Functional architecture of pancreatic islets identifies a population of first responder cells that drive the first-phase calcium response. PLOS Biol. 20, e3001761 (2022). 36099294 10.1371/journal.pbio.3001761 PMC9506623

[64] D. Korosak, M. Jusup, B. Podobnik, A. Stozer, J. Dolensek, P. Holme, M. S. Rupnik, Autopoietic influence hierarchies in pancreatic β cells. Phys. Rev. Lett. 127, 168101 (2021). 34723613 10.1103/PhysRevLett.127.168101

[65] L. S. Satin, Q. Zhang, P. Rorsman, “Take me to your leader”: An electrophysiological appraisal of the role of hub cells in pancreatic islets. Diabetes 69, 830–836 (2020). 32312899 10.2337/dbi19-0012 PMC7171959

[66] R. K. Benninger, M. Zhang, W. S. Head, L. S. Satin, D. W. Piston, Gap junction coupling and calcium waves in the pancreatic islet. Biophys. J. 95, 5048–5061 (2008). 18805925 10.1529/biophysj.108.140863 PMC2586567

[67] R. K. Benninger, T. Hutchens, W. S. Head, M. J. McCaughey, M. Zhang, S. J. Le Marchand, L. S. Satin, D. W. Piston, Intrinsic islet heterogeneity and gap junction coupling determine spatiotemporal Ca^2+^ wave dynamics. Biophys. J. 107, 2723–2733 (2014). 25468351 10.1016/j.bpj.2014.10.048 PMC4255172

[68] J. Dolenšek, A. Stožer, M. Skelin Klemen, E. W. Miller, M. Slak Rupnik, The relationship between membrane potential and calcium dynamics in glucose-stimulated beta cell syncytium in acute mouse pancreas tissue slices. PLOS ONE 8, e82374 (2013). 24324777 10.1371/journal.pone.0082374 PMC3855743

[69] J. V. Rocheleau, M. S. Remedi, B. Granada, W. S. Head, J. C. Koster, C. G. Nichols, D. W. Piston, Critical role of gap junction coupled K_ATP_ channel activity for regulated insulin secretion. PLOS Biol. 4, e26 (2006). 16402858 10.1371/journal.pbio.0040026 PMC1334237

[70] N. L. Farnsworth, R. K. Benninger, New insights into the role of connexins in pancreatic islet function and diabetes. FEBS Lett. 588, 1278–1287 (2014). 24583073 10.1016/j.febslet.2014.02.035 PMC4004767

[71] S. Speier, A. Gjinovci, A. Charollais, P. Meda, M. Rupnik, Cx36-mediated coupling reduces β-cell heterogeneity, confines the stimulating glucose concentration range, and affects insulin release kinetics. Diabetes 56, 1078–1086 (2007). 17395748 10.2337/db06-0232

[72] J. Fernandez, M. Valdeolmillos, Glucose-dependent stimulatory effect of glucagon-like peptide 1(7-36) amide on the electrical activity of pancreatic beta-cells recorded in vivo. Diabetes 48, 754–757 (1999). 10102691 10.2337/diabetes.48.4.754

[73] G. G. Holz, W. M. Kuhtreiber, J. F. Habener, Pancreatic beta-cells are rendered glucose-competent by the insulinotropic hormone glucagon-like peptide-1(7-37). Nature 361, 362–365 (1993). 8381211 10.1038/361362a0 PMC2916679

[74] R. Gao, S. Acreman, H. Dou, J. Ma, C. Miranda, R. Zhao, M. T. Dickerson, A. Tarasov, Q. Zou, M. Gironella-Torrent, J. Tolö, A. Clark, R. Gao, Y. De Marinis, D. A. Jacobson, J. Camunas-Soler, T. Yang, P. Rorsman, Q. Zhang, Antecedent hypoglycaemia impairs glucagon secretion by enhancing somatostatin-mediated negative feedback control. Nat. Metab. 8, 159–176 (2026). 41530286 10.1038/s42255-025-01422-7

[75] H. Dou, C. Miranda, J. Tolö, C. Santos, R. Gao, N. R. Gandasi, T. G. Hill, L. Kothegala, A. I. Tarasov, Q. Zhang, P. Rorsman, Metabolic and paracrine heterogeneity of pancreatic glucagon-secreting α-cells. Diabetes 74, 2307–2321 (2025). 40184031 10.2337/db24-1053

[76] E. Ipp, R. E. Dobbs, A. Arimura, W. Vale, V. Harris, R. H. Unger, Release of immunoreactive somatostatin from the pancreas in response to glucose, amino acids, pancreozymin-cholecystokinin, and tolbutamide. J. Clin. Invest. 60, 760–765 (1977). 330567 10.1172/JCI108829 PMC372422

[77] M. Phillip, A. Kowalski, T. Battelino, Type 1 diabetes: From the dream of automated insulin delivery to a fully artificial pancreas. Nat. Med. 30, 1232–1234 (2024). 38448742 10.1038/d41591-024-00013-5

[78] L. Olcomendy, L. Cassany, A. Pirog, R. Franco, E. Puginier, M. Jaffredo, D. Gucik-Derigny, H. Rios, A. Ferreira de Loza, J. Gaitan, M. Raoux, Y. Bornat, B. Catargi, J. Lang, D. Henry, S. Renaud, J. Cieslak, Towards the integration of an islet-based biosensor in closed-loop therapies for patients with type 1 diabetes. Front. Endocrinol. 13, 795225 (2022). 10.3389/fendo.2022.795225 PMC9072637 35528003

[79] P. L. Herrera, Adult insulin- and glucagon-producing cells differentiate from two independent cell lineages. Development 127, 2317–2322 (2000). 10804174 10.1242/dev.127.11.2317

[80] F. Thorel, V. Népote, I. Avril, K. Kohno, R. Desgraz, S. Chera, P. L. Herrera, Conversion of adult pancreatic α-cells to β-cells after extreme β-cell loss. Nature 464, 1149–1154 (2010). 20364121 10.1038/nature08894 PMC2877635

