# Supplementary material for: Pancreatic α cells are required for nutrient homeostasis by regulating dynamic β cell networks in islets
Source: Sci Adv. 2026 Jul 3;12(27):eaea9045. doi: 10.1126/sciadv.aea9045 (PMC13330822; doi:10.1126/sciadv.aea9045)
Supplement: Supplementary file 1 — Figs. S1 to S7 [file sciadv.aea9045_sm.pdf]

Science Advances
AAAS

Supplementary Materials for

Pancreatic α cells are required for nutrient homeostasis by regulating dynamic β cell networks in islets

Marie Lallouet *et al.*

Corresponding author: Jochen Lang, jochen.lang@u-bordeaux.fr

Sci. Adv. **12**, eaea9045 (2026)
DOI: 10.1126/sciadv.aea9045

This PDF file includes:

Figs. S1 to S7

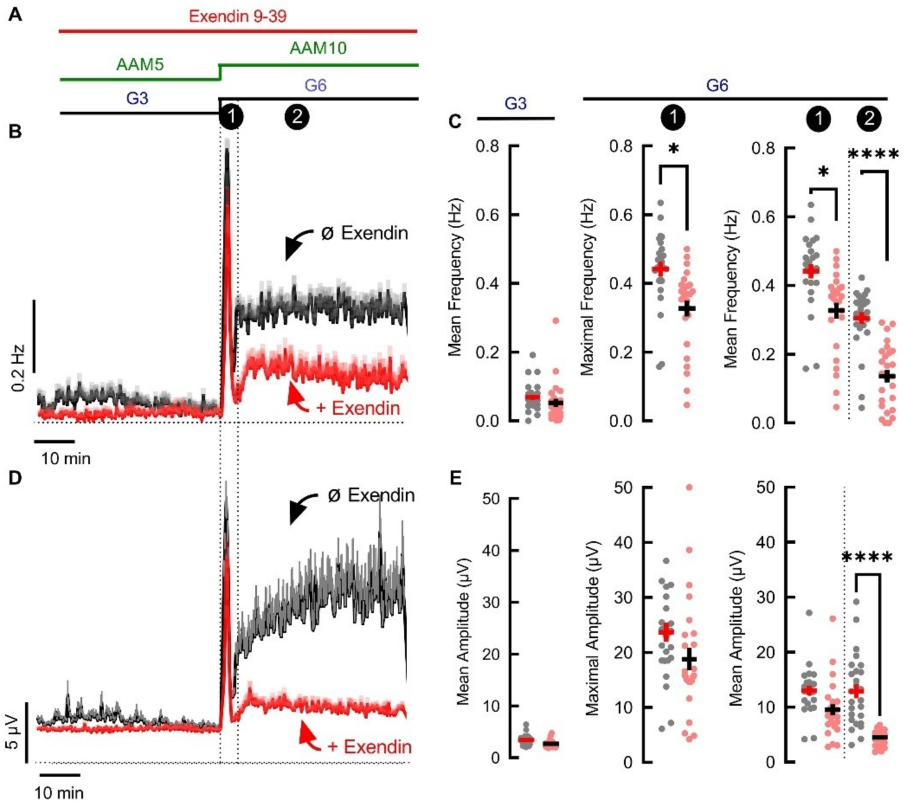

Fig. S1: Effect of the GLP-1 antagonist Exendin 9-39 on glucose and amino-acid evoked electrical activity in WT islets. (**A**) Incubation scheme, glucose (3 mM, G3; 6 mM G6), amino acids (5 mM, AA5; 10 mM AA 10) and Exendin 9-39 (1 nM). Black, no exendin 9-39; red, in the presence of exendin 9-39. (**B**) Mean slow potential frequencies (±SEM). 1st and 2nd phases are indicated (❶,❷). N= 2, n=26. (**C**) Statistics of frequencies. (**D, E**) Mean slow potential amplitudes (±SEM) and statistics. 2-way ANOVA and Kruskal; *, **, ***, ****, 2p<0.05, <0.01, <0.001, <0.0001.

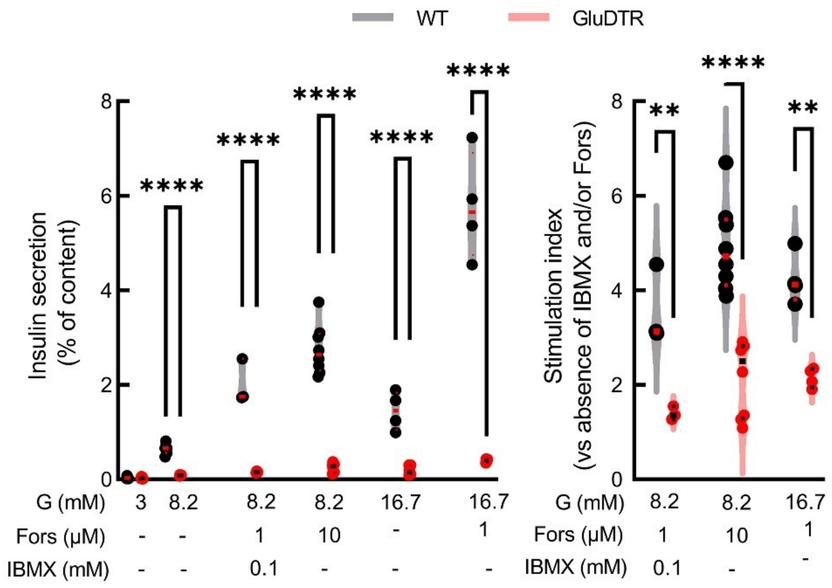

Fig. S2: Effect of IBMX and/or forskolin on glucose and amino-acid stimulated in WT and GluDTR islets. (**A**) insulin secretion at different glucose (G) concentrations in the absence or presence of IBMX and/or forskolin. N=3-8 (**B**) Stimulation indices for indicated conditions versus those without IBMX and or forskolin. 2-way ANOVA and Tukey; **, ****, 2p<0.01, <0.0001.

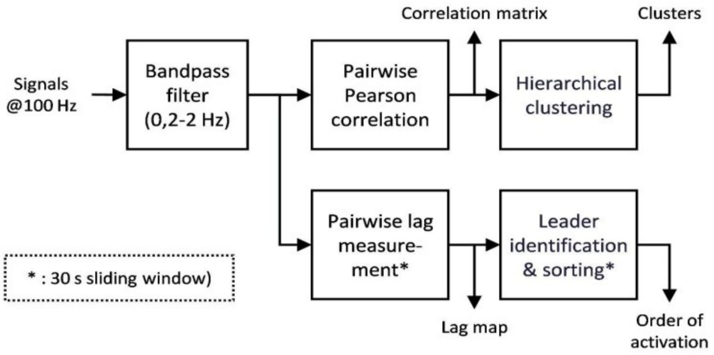

Fig. S3: Scheme of data analysis of HD MEA data

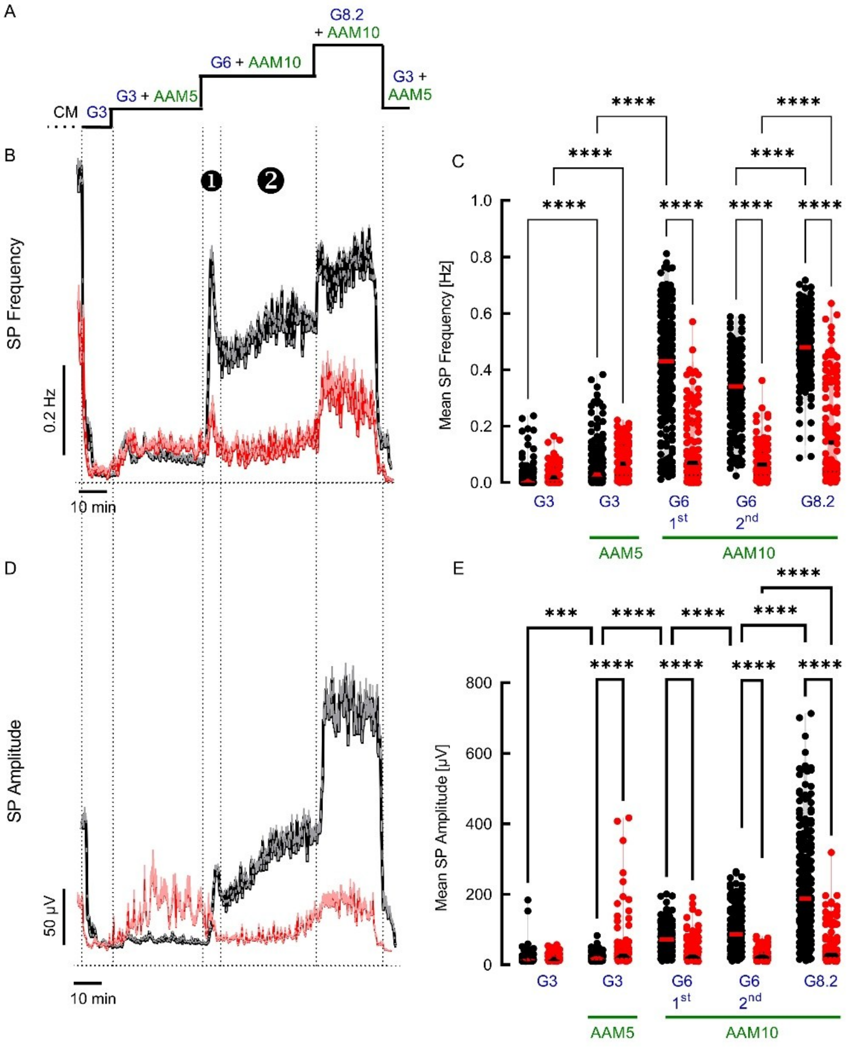

Fig. S4: Recordings of WT and GluDTR islets on high-density MEAs. (**A**) Incubation scheme. **(B)** Effect of glucose (3 mM, G3; 8.2 mM G8.2) and amino acids (5 mM, AA5; 10 mM AA 10) on mean slow potential frequencies (±SEM). 1st and 2nd phases are indicated (❶,❷). (**C**) Statistics of mean frequencies. (**D)** Effect of glucose and amino acids on mean slow potential amplitudes (±SEM). (❶,❷). (**E**) Statistics of mean amplitudes. 3 animals and 11 islets total; 2-way ANOVA and Tukey; ***, ****, 2p <0.001, <0.0001.

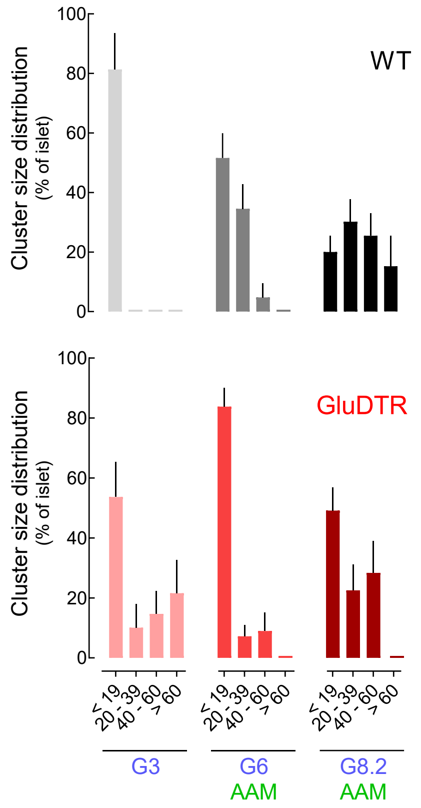

Fig. S5: Histogram of cluster size distribution. Islets were exposed to 3 mM glucose (G3), 6 mM glucose and amino acids (G6 AAM) or 8.2 mM glucose and amino acids (G8.2 AAM) in WT or GluDTR islets. (**A**) WT; (**B**) GluDTR. N=3 animals, n=11 islets for each condition.

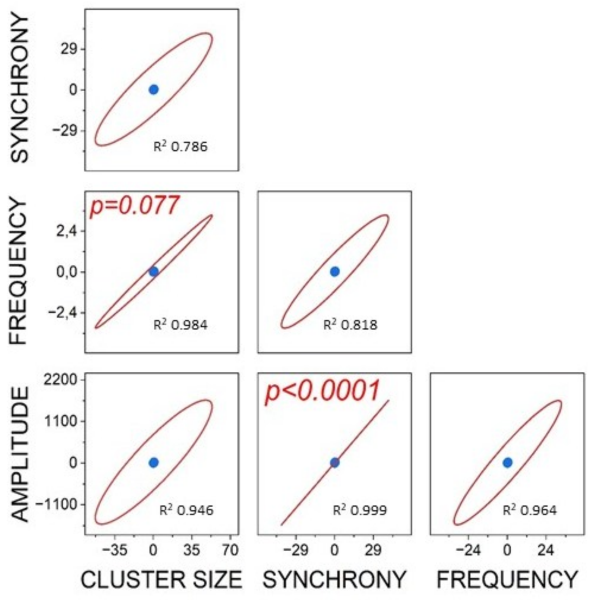

Fig. S6: Correlations between synchrony, cluster size, frequency and amplitude. The data from HD-MEA recordings in terms of mean frequency or mean amplitude during G8.2 stimulation (in the presence of amino acids) of wild type islets were used. Pearson correlations with center points and 99% confidence intervals (red line), R^2 and relevant p values are given; Data from 3 animals, and 11 islets total as in Fig. 5.

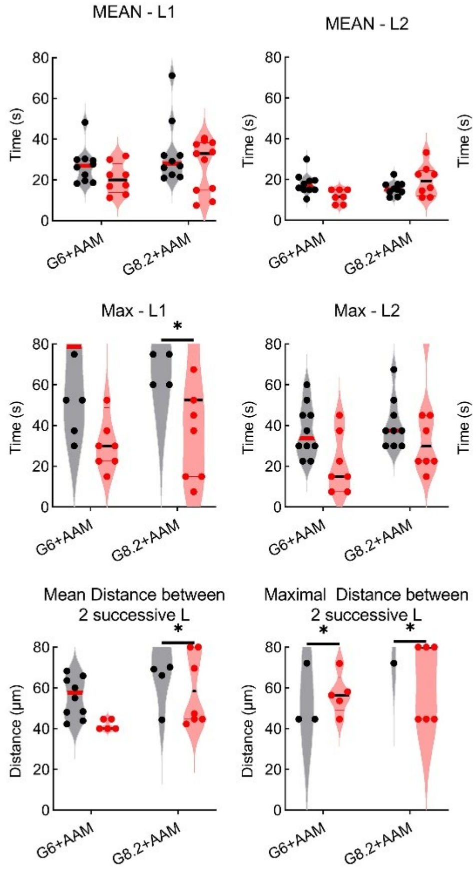

Fig. S7: Temporal stability of leader regions and distances between successive leader regions. (**A**) mean stability in seconds for first, second, third and subsequent leader regions as well as all leader regions. Black, wild type; red, GluDTR. (**B**) Maximal stability for leader regions. (**C**) Mean and maximal distance between two successive leader.
